# Comprehensive study of volatile compounds and transcriptome data providing genes for grape aroma

**DOI:** 10.1186/s12870-023-04191-1

**Published:** 2023-03-31

**Authors:** Yongzhou Li, Liangliang He, Yinhua Song, Peng Zhang, Doudou Chen, Liping Guan, Sanjun Liu

**Affiliations:** Zhengzhou Fruit Research Institute, Chinese Academy of Agricultural Sciences, Zhengzhou, 450009 People’s Republic of China

**Keywords:** Aroma, Grapes, SPME-GC-MS, Transcriptome, Volatile compounds

## Abstract

**Background:**

Fruit aroma is an important quality with respect to consumer preference, but the most important aroma compounds and their genetic regulatory mechanisms remain elusive.

**Results:**

In this study, we qualitatively analysed volatile compounds in the pulp and skin of five table grape cultivars with three aroma types (muscat, strawberry, and neutral) using solid-phase microextraction gas chromatography/mass spectrometry. We identified 215 aroma compounds, including 88 esters, 64 terpenes, and 29 alcohols, and found significant differences in the number of compounds between the pulp and skin, especially for terpenes. Skin transcriptome data for the five grape cultivars were generated and subjected to aroma compound-gene correlation analysis. The combined transcriptomic analysis and terpene profiling data revealed 20 candidate genes, which were assessed in terms of their involvement in aroma biosynthetic regulation, including 1 *VvCYP* (VIT_08s0007g07730), 2 *VvCCR* (VIT_13s0067g00620, VIT_13s0047g00940), 3 *VvADH* (VIT_00s0615g00010, VIT_00s0615g00030, VIT_ 00s0615g00020), and 1 *VvSDR* (VIT_08s0040g01200) in the phenylpropanoids synthesis pathway, and 1 *VvDXS* (VIT_05s0020g02130) and 6 *VvTPS* (VIT_13s0067g00370, Vitis_vinifera_newGene_3216, VIT_13s0067g00380, VIT_13s0084g00010, VIT_00s0271g00010, and VIT_13s0067g00050) in the methylerythritol phosphate pathway (involved in the production and accumulation of aromatic compounds). Additionally, 2 *VvMYB (VIT_17s0000g07950, VIT_03s0063g02620)* and 1 *VvGATA* (VIT_15s0024g00980) transcription factor played important regulatory roles in the accumulation of key biosynthetic precursors of these compounds in grapes. Our results indicated that downstream genes, specifically 1 *VvBGLU* (VIT_03s0063g02490) and 2 *VvUGT* (VIT_17s0000g07070, VIT_17s0000g07060) are involved in regulating the formation and volatilization of bound compounds in grapes.

**Conclusions:**

The results of this study shed light on the volatile compounds and “anchor points” of synthetic pathways in the pulp and skin of muscat and strawberry grapes, and provide new insight into the regulation of different aromas in grapes.

**Supplementary Information:**

The online version contains supplementary material available at 10.1186/s12870-023-04191-1.

## Background

Aroma is one of the most important indicators of fruit quality. Aroma involves both taste and smell. Aroma is influenced by a variety of factors that are important for breeding [1]. Volatile compounds play a vital role in fruit aroma; they are responsible for the primary aroma of alcohols, esters, aldehydes, terpenes, etc. However, only a few of these substances play a decisive role in fruit flavour and aroma [1, 2].

Pathways for the synthesis of aromatic substances in plant fruits include fatty acid metabolism, amino acid metabolism, terpene metabolism, and monosaccharide and glycoside metabolism. The aromatic substances in fruit are mainly derived from saturated and unsaturated fatty acids, which are metabolized by the aerobic pathway of fatty acid metabolism (including lipoxygenase and β-oxidation pathways) to form straight-chain fatty alcohols, aldehydes, ketones, acids, and esters [3]. Amino acids such as valine, leucine, isoleucine, alanine and cysteine are direct precursors of aromatic substances in fruit, and their metabolism produces large amounts of alcohols, aldehydes, esters, and carbonyls [3]. There are two types of amino acids. The first type, aliphatic amino acids, are converted into branched-chain aliphatic alcohols, aldehydes, ketones, and esters in the plant. The second type, aromatic amino acids, are the main source of volatile phenols, ethers, and certain aromatic ring-containing compounds. The first step in terpene synthesis is the synthesis of isopentenyl pyrophosphate and its isomer dimethyl allyl pyrophosphate. There are two main pathways for the synthesis of isopentane pyrophosphate: the mevalonate pathway and 2 C-methyl-D-erythritol-4-phosphate pathway (MEP) pathway [4]. In addition, the monosaccharides in fruit are used not only as flavouring substances, but also as precursors for aroma substances [5–7].

Horticultural research has shown that fruit flavour varies greatly depending on the species of fruit tree. Generally, the aromatic composition of fruit is “purer” than that of other foodstuffs, and most have a natural (light or strong) aromatic smell [8, 9]. More than 300 aromatic components have been detected in kiwifruit, and the flavour intensity of the three main volatile components, ethyl butyrate, €-2-hexenal, and hexanal, has been evaluated; notably, hexanal a€(E)-2-hexenal have a prominent effect on the flavour quality of kiwifruit [10]. More than 120 aromatic substances have been detected in pear fruit, most of which are esters (propyl acetate, ethyl acetate, etc.), aldehydes (hexanal, nonanal, etc.), alcohols (ethanol, propanol, etc.), alkanes (hexadecane, etc.), and olefins (alpha-farnes, etc.). More than 100 aromatic compounds have been identified in peach fruit, and C6 aldehydes and alcohols with a greenish aroma are the main aromatic compounds of both peach and nectarine. Aroma analysis of ‘Okubo’ peach fruit revealed that esters such as hexyl acetate and (Z)-3-hexenyl acetate are key aroma enhancers affecting peach flavour quality; they have the highest percentages of ester volatiles [11]. Many studies have shown that γ-decalactone and δ-decalactone are the most important lactones in peach fruit and play an important role in the unique aroma of peach fruit. Apples contain over 300 volatile compounds and have high levels of esters. The volatile compounds vary considerably among cultivars, and the characteristic aroma of Fuji apples is mainly derived from ethyl butyrate, ethyl acetate and hexyl acetate. The odour of ‘Elstar’ apples is mainly attributed to ethyl butyrate and ethyl butyrate, while that of ‘Cox Orange’ apples is attributed to ethyl butyrate, acetaldehyde, 2-methylbutanol, and methyl propionate [12]. Apricots contain > 200 volatile compounds, of which the 6 most important are hexanal, (E)-2-hexenal, linalool, 1-hexanol, ethyl caprylate, and hexyl acetate); previous studies have shown that these compounds are responsible for apricot flavour [13].

Grape (*Vitis vinifera* L.) is one of the most widely cultivated and economically important fruit crops. Because a rich aroma is essential for table and wine grape quality, numerous studies have focused on aroma characteristics. At present, more than 2,000 volatile components have been identified in grapes [14]. Esters, alcohols, aldehydes, ketones, and volatile terpenes are among the key components of grape aroma and can be classified based on their characteristics and taste. Table grape aromas can be divided into non-, strawberry-, muscat-, and fox aromas. For ‘neutral’ grapes, the terpene and ester amounts are low and mainly comprise aldehydes and alcohols, such as hexanal and 2-hexenal, which give rise to less intense aromas. Strawberry-scented grapes are mainly American varieties and their hybrids; their characteristic compounds are mainly esters, i.e., ethyl acetate, ethyl butyrate, ethyl 2-butenoate, and ethyl 2-hexenoate [15]. In addition to esters, 4-hydroxy-2,5-dimethyl-3(2 H) furanone is also an important volatile substance for strawberry aroma [16]. The main volatile compounds of muscat-scented grapes are sesquiterpenes and monoterpenes, which are low molecular weight compounds. In particular, monoterpenes are abundant in muscat-scented cultivars [17]. Ethyl benzoate is the characteristic aroma substance of the fox aroma type, which is characterized by fruity, sweet, and creamy aromas [8, 18]. Canosa [19] et al. experimented with five red grape cultivars and found that the most abundant aromatic substances were free C_6_ compounds and alcohols. Rocha [20] et al. studied four white grape cultivars and found that terpenes, C13-nor isoprene derivatives, and alcohols were the only abundant compounds.

With the rapid development of analytical techniques, such as solid-phase microextraction gas chromatography/mass spectrometry (SPME-GC-MS), our understanding of volatile compounds has increased dramatically. These volatiles, acting as free or bound chemicals, contribute to fruit aroma. However, only volatiles with odour activity values (OAVs; OAV = volatile contents/order threshold) > 1 are considered to contribute to the characteristic aroma. The aroma of grapes is complex, of which it is mainly known for its muscat and strawberry. The aroma of muscat grapes has been extensively studied; however, little attention has been paid to strawberry aroma or differences in aroma substances between strawberry- and muscat-scented grapes. Compared with other fruits, the genetic mechanisms underlying the biosynthesis and metabolism of grape berries have only just begun to be elucidated. The compounds underlying the different aromas of grape cultivars have yet to be fully resolved, and the regulatory genes also remain to be elucidated. To fully evaluate the aroma characteristics of strawberry and muscat aromas, the volatile compounds and regulatory genes must be analysed in more detail.

In this study, we evaluated volatile compounds in the pulp and skin of five table grapes with two aroma types, muscat aroma (‘Shine Muscat’, ‘Midnight Beauty’, and ‘Centennial Seedless’) and strawberry aroma (‘Summer Black’), as well as a neutral grape (‘Victoria’), using SPME-GC-MS. The unscented ‘Victoria’ was used as a control to investigate the specific composition of each aroma type. The skins of the grapes with different aromas were subjected to transcriptome sequencing. Association analysis of volatile compounds with differentially expressed genes (DEGs) revealed by transcriptome sequencing was used to identify candidate genes after controlling for the different aromas of the grapes. The results from this provide insight into the main substances in muscat and strawberry grapes and lay a foundation for further investigations of the genetic mechanisms of grape aroma.

## Results

### Basic physicochemical parameters of the berry

**Fig. 1:**
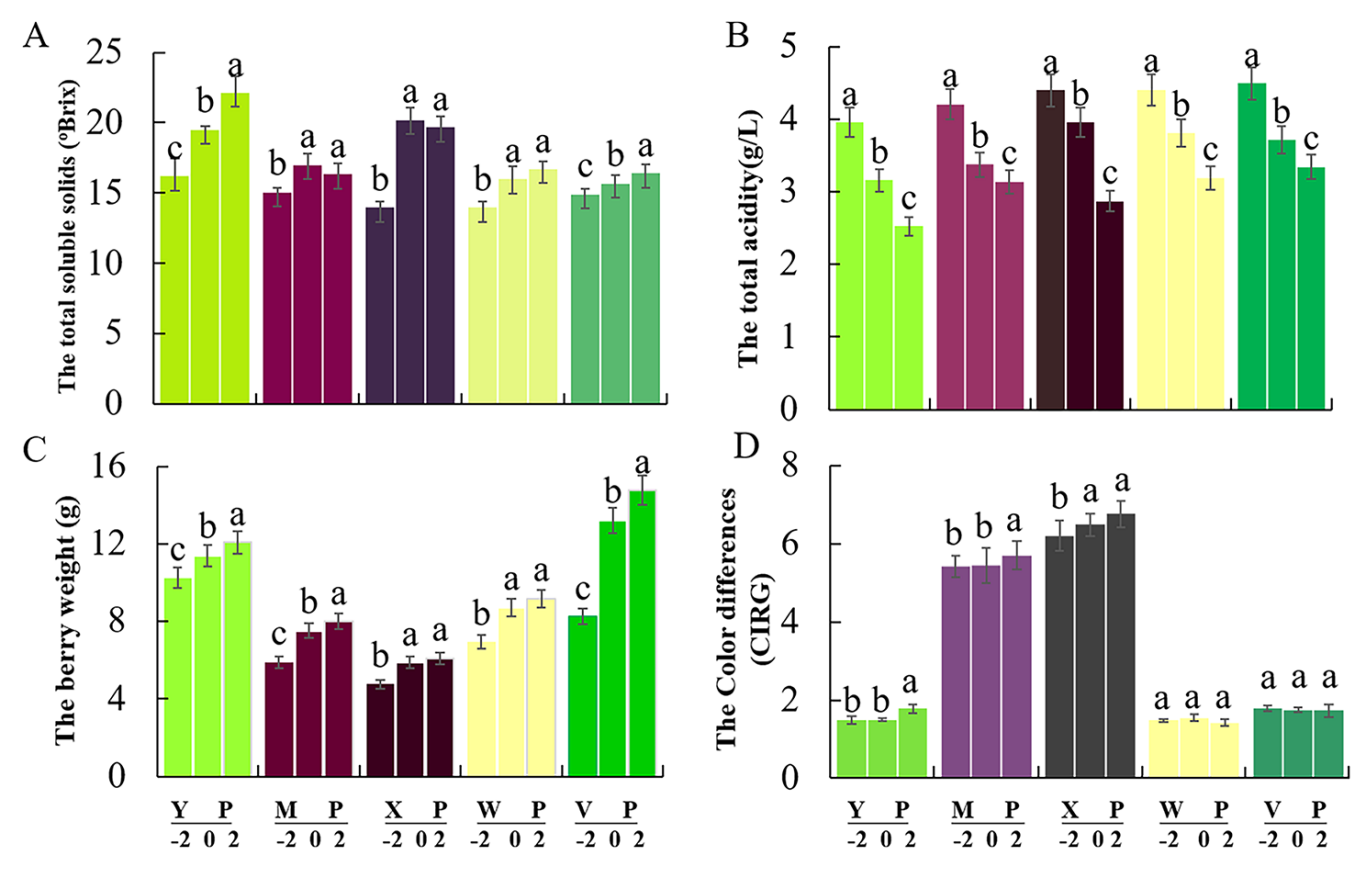
Physicochemical changes during five grape cultivars development. **(A)** The total soluble solids(°BRIX); **(B)** The total acidity (g/L); **(C)** The berry fresh weight (g); **(D)** The skin color index of red grape during three berry developmental stages from − 2 to 2 weeks ripen-stage (wrs) were analyzed. XP stands for ‘Summer Black’, YP stands for ‘Shine Muscat’, MP stands for ‘Midnight Beauty, WP stands for ‘Centennial Seedless’ and VP stands for ‘Victoria’. Mean ± standard error (n = 5). Different letters indicate significant differences (Duncan’s multiple range test, P ≤ 0.05)

Basic parameters of the grapes, including the TSSs (A), TA (B), berry weight (C), and skin CIRG (D) were measured (Fig. 1). The measurements revealed significant differences in the dynamics of grape fruit ripening among grape cultivars. TSSs and berry weight exhibited an increasing trend over the course of ripening, i.e., from − 2 wrs to 0 wrs to 2 wrs, whereas TA displayed a decreasing trend during berry development. The skin colour index was also correlated with the ripeness of grapes. Moreover, YP, WP, and VP all showed a lime-green colour; however, YP and WP both had a muscat aroma, whereas VP did not have any aroma. MP and XP both showed a dark red colour, but with a muscat and strawberry aroma, respectively. By calculating and observing the colour index of the skin, we found that the aroma of the grapes was not directly related to the skin colour. According to the OIV resolution VITI 1/2008, table grapes are considered ripe with a TSS ≥ 16 °Brix. Overall, these results indicated that all of the grape berries reached maturity at the appropriate time and their aroma types were typical.

### Quantification of volatile compounds

**Fig. 2:**
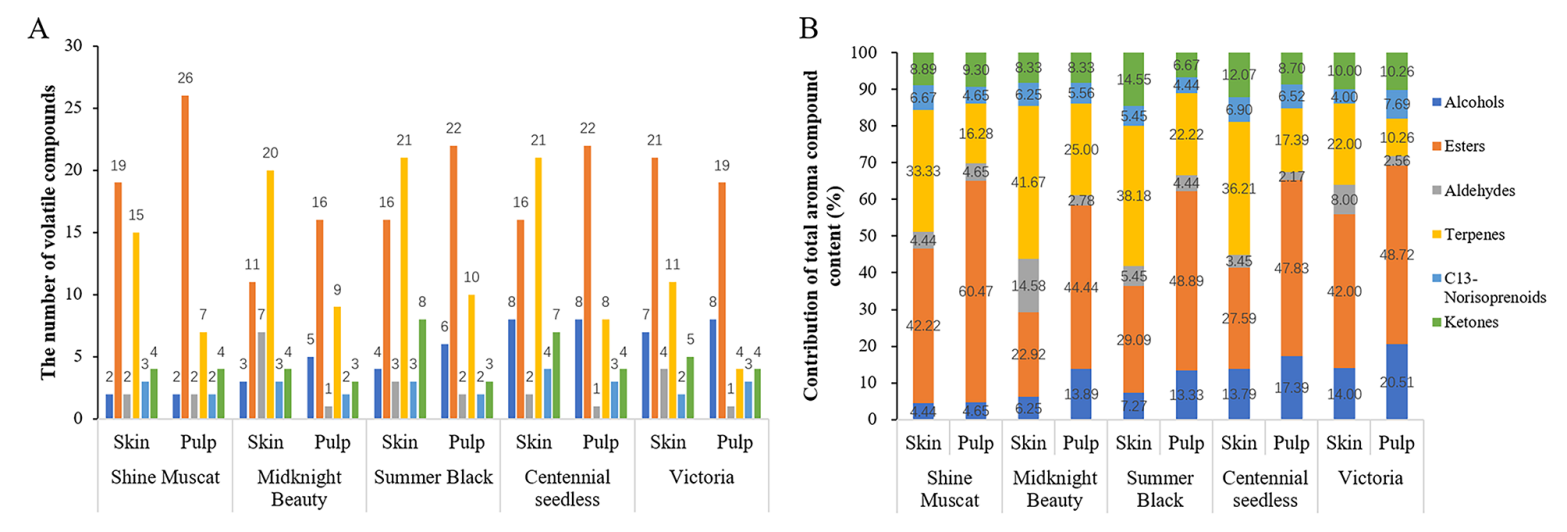
Composition and quantitative statistics of volatile compounds in skin and pulp of five grape cultivars. **(A)** Types and amounts of volatile compounds in the skins and pulp of the five grape cultivars. **(B)** Contribution ratio of total aromatic substance content in skins and pulp

The volatile aroma components in the five cultivars of cultivated grapes with different aromas were analysed by SPME-GC-MS, and ion flow diagrams of the volatile aroma components in the skins and pulps of the grapes were obtained (Figure S1 and S2). A total of 215 volatile compounds, including 112 in pulp and 151 in skin, were identified in these five grape cultivars. There were significant differences in the number of volatile compounds among the grape cultivars, and the same volatile compounds appeared to be present in both the skin and pulp (Figure S3). However, the number of volatile compounds in the skin was significantly higher than in the pulp (45–55 and 36–46 compounds, respectively, for each cultivar). Among these compounds, esters and terpenes were present in the greatest numbers, indicating major contributions to aroma. The number of volatile compounds in the skin and pulp of the strawberry aroma cultivar (‘Summer Black’) was higher than in other cultivar aroma types (Fig. 2).

The total volatile compound contents exhibited significant differences between the skin and pulp in the five grape cultivars (Supplementary tables S1 and S2). The different volatile compound contents did not show a uniform increasing or decreasing trend over the three periods (− 2, 0, and 2 wrs), unlike the total contents of each class of volatiles in skin. In the skin of ‘Shine Muscat’, the ester and aldehyde contents decreased from − 2 wrs to 2 wrs, whereas the terpene content increased. In the skin of ‘Midnight Beauty’, the aldehydes and terpene contents were highest at the ripening stage. In the skin of ‘Summer Black’, which has a strawberry aroma, the alcohol and ester contents decreased, and aldehyde and ketone amounts were highest at the ripening stage. In the skins of the unscented grape ‘Victoria’, the alcohol, aldehyde, terpene, and C13-norisoprenoid contents did not vary significantly among the three periods.

In the pulp of ‘Shine Muscat’, the alcohol, terpene, and ketone contents were highest in the ripening stage, and the ester content increased from − 2 wrs to 2 wrs. In the pulp of ‘Midnight Beauty’, there was no significant change in the content of volatile compounds. The pulp of ‘Summer Black’ showed higher contents of aldehydes, terpenes, and ketones in the ripening stage, and the ester content increased from − 2 wrs to 2 wrs. In the flesh of ‘Victoria’, the alcohol and aldehyde contents decreased from − 2 wrs to 2 wrs; the ester and terpene contents were highest at maturity, and the content of the remaining substances did not change significantly.

The volatile compound contents in the skin were significantly higher than those in pulp (specifically, 98.19–764.04 µg.kg^− 1^ in the skin and a maximum of 67.54 µg.kg^− 1^ in the pulp). Among these compounds, terpenes and aldehydes were dominant in the skin of strawberry and muscat cultivars, whereas ester and aldehydes were the main volatiles in the skin of the neutral cultivar. Notably, terpenes and esters were the dominant compounds in the pulp of strawberry and muscat cultivars. The differences in composition and content of volatile compounds between the skin and pulp suggest that the aroma may be synthesized and metabolized in different ways in these compartments.

### Identification of volatile compounds

**Fig. 3:**
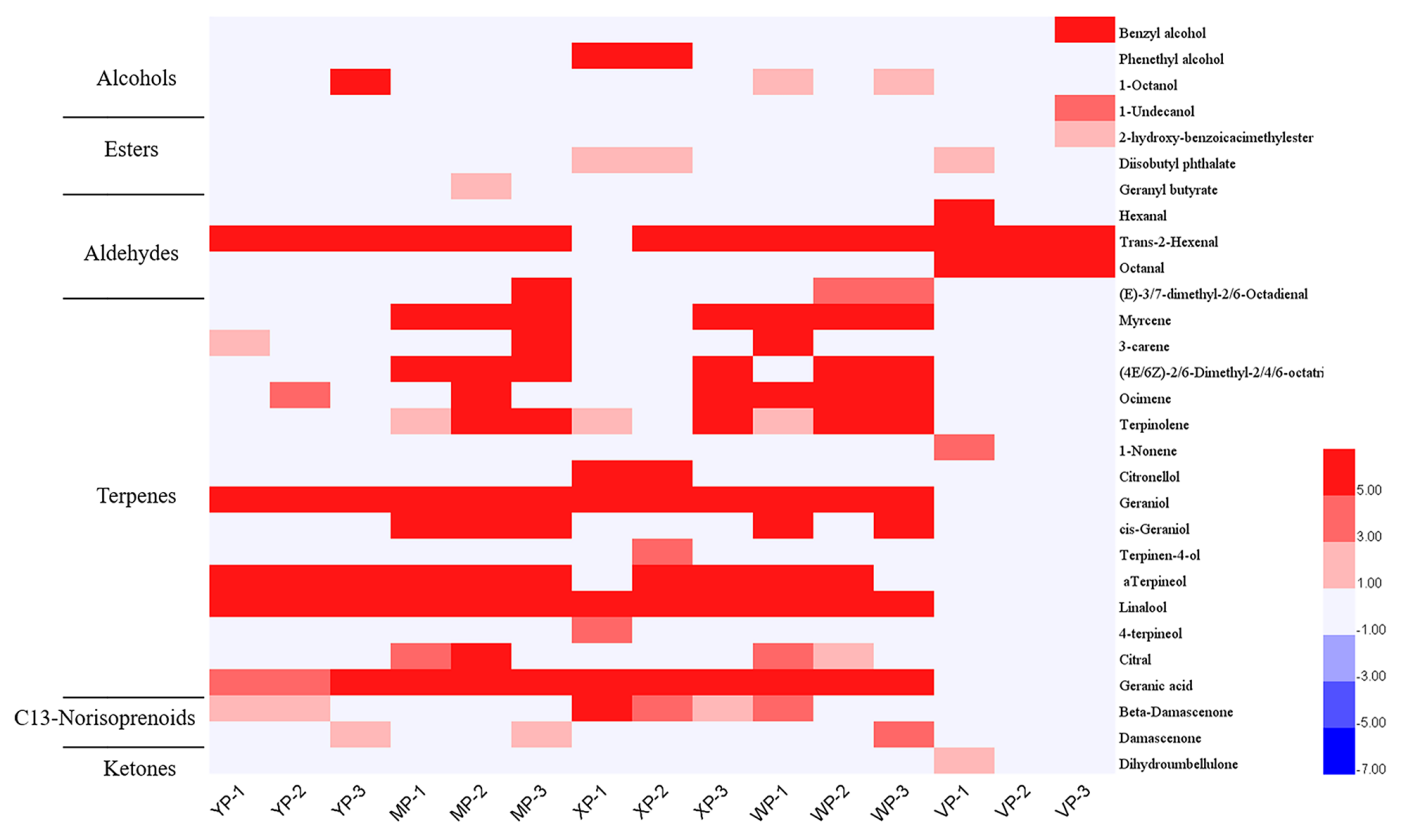
Comparative analysis of heat maps of aroma compounds in skins of five grape cultivars. YP stands for ‘Shine Muscat’, MP stands for ‘Midnight Beauty, XP stands for ‘Summer Black’, WP stands for ‘Centennial Seedless’ and VP stands for ‘Victoria’. 1,2 and 3 indicate − 2wrs, 0wrs and 2wrs respectively

**Fig. 4:**
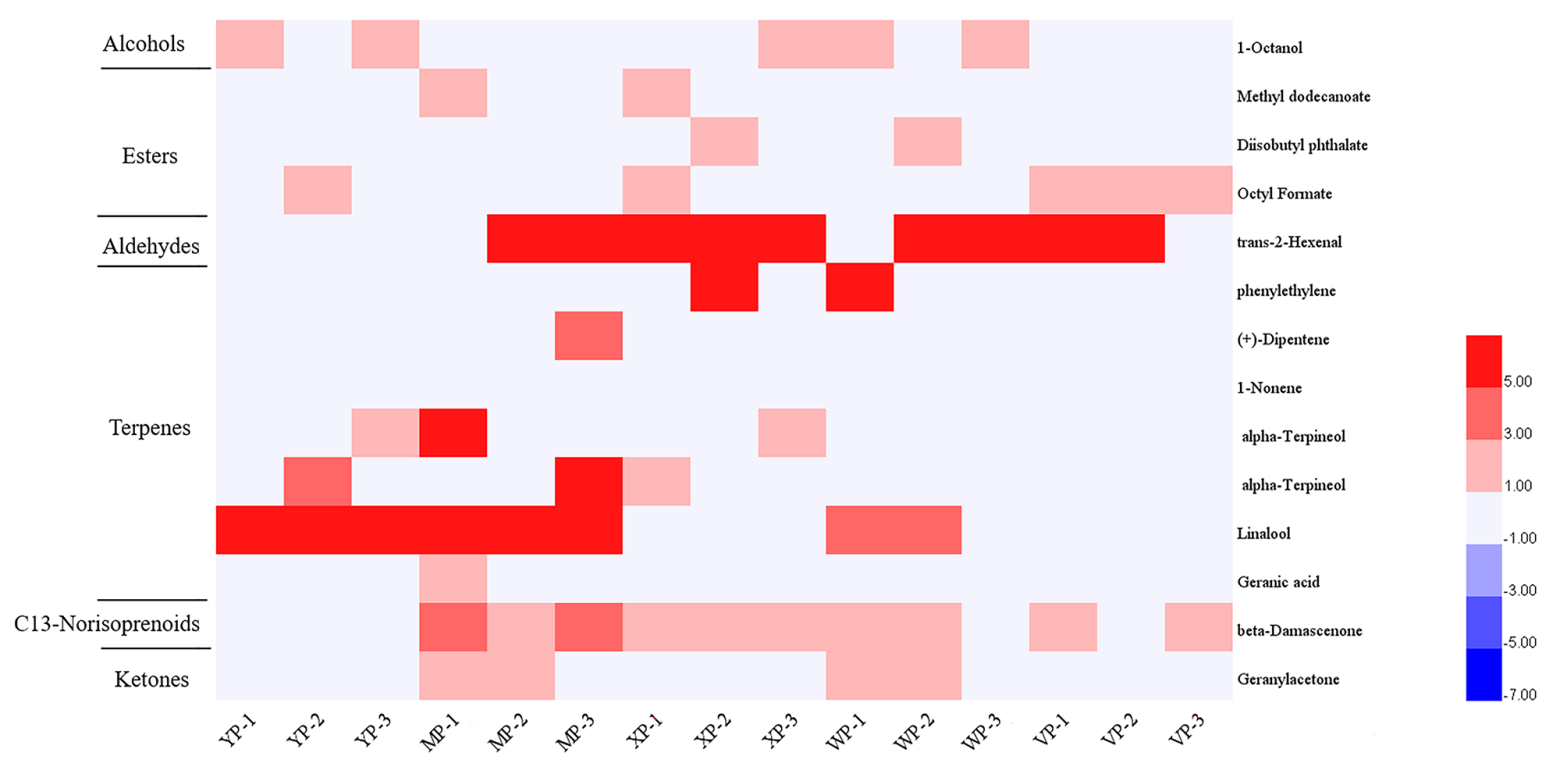
Comparative analysis of heat maps of aroma compounds in pulps of five grape cultivars. YP stands for ‘Shine Muscat’, MP stands for ‘Midnight Beauty, XP stands for ‘Summer Black’, WP stands for ‘Centennial Seedless’ and VP stands for ‘Victoria’. 1,2 and 3 indicate − 2wrs, 0wrs and 2wrs respectively

For the analysis of grapes with different aromas, the OAV is commonly used to determine the contribution of grape fruit aroma substances to the aroma characteristics of grapes. It is generally accepted that aromatic substances with an OAV > 1 contribute to the overall aroma of the grape, and that the greater the OAV, the higher the overall contribution, as demonstrated in the Verdell grape, for example, a total of 87 free aromatic species and 24 aromatic substances with OAV > 1 were found in Verdell grapes, which play an important role in the aroma of the grapes [21]. In this experiment, a total of 65 free aroma species were measured, and 29 and 14 aroma-active compounds (OAVs > 1) were detected in the skin and pulp of the five grape cultivars, respectively. Figures 3 and 4 show the cluster analysis results and associated heat map, respectively.

Terpenes, C13-norisoprenoids, and esters were the main contributors to aroma, and their OAVs were higher in the skins than pulp. Trans-2-hexenal was the active aldehyde, while geranic acid and geraniol were the active terpenoids that contributed to the grape flavour. Among the aldehydes, the OAV of trans-2-hexenal was significantly higher in the skins of grapes with aromas (Shine Muscat, Midnight Beauty, Summer Black, and Centennial Seedless) than in Victoria (neutral). In the pulp, however, there was no significant difference in the extracted compounds with and without aromas.

In the skins, terpenes (with the exception of 1-nonene) were detected only in aromatic grapes, in widely varying amounts; specifically, citronellol, 4-terpineol, and terpinen-4-ol were detected only in Summer Black, which has a strawberry aroma. 3-carene, cis-geraniol, and citral were only detected in the muscat-fragranced species. Hexanal and octanal in the aldehyde species, benzyl alcohol and 1-undecanol in the alcohols, and 2-hydroxy-benzoicacimethylester in the esters were detected in the neutral grapes. In addition, 1-octanol, geranyl butyrate, (E)-3,7-dimethyl-2,6-octadienal, and damascene were detected only in the muscat-scented cultivars.

In the pulp, very few threshold aroma components were detected. Terpenes and ketones were found only in the aromatic grapes. Dipentene, 1-nonene, linalool, geranic acid (among the terpenes), and geranyl acetone (among the ketones) were detected only in the muscat cultivars. Among the esters, with the exception of octyl formate, which was detected only in the aromatic grapes, the amount of octyl formate did not vary significantly between the different aromas; methyl dodecanoate and diisobutyl phthalate were detected only in the aromatic cultivars, and their OVA values were small. Beta-damascenone belonged to ‘C13-norisoprenoids’ and 1-Octanol belonged to the ‘alcohols’, but was detected in all five grape cultivars. Moreover, the differences were not significant, so it was not a major contributor to the differences in pulp aroma. Among these substances, some had an OVA value < 3 still contributed significantly to the aroma of the grape berries, due to their low threshold values. The alcohols and aldehydes presented with little odour due to their high thresholds; for example, trans-2-hexenal was the active aldehyde in Victoria, but had neutral. The differences in these components contributed to the fruity aroma of the cultivars.

### Multivariate analysis

There was no clear pattern in the volatile content of the various aroma types, and few volatiles contributed to the aroma of the fruit. PCA is a multivariate analysis technique used to downscale datasets to resolve the correlations between variables and samples [22, 23]. As the volatile substances were found in much higher quantities in the skin of the fruit than in the pulp in our experiment, the volatile substances in the skin were subjected to PCA analysis.

**Fig. 5:**
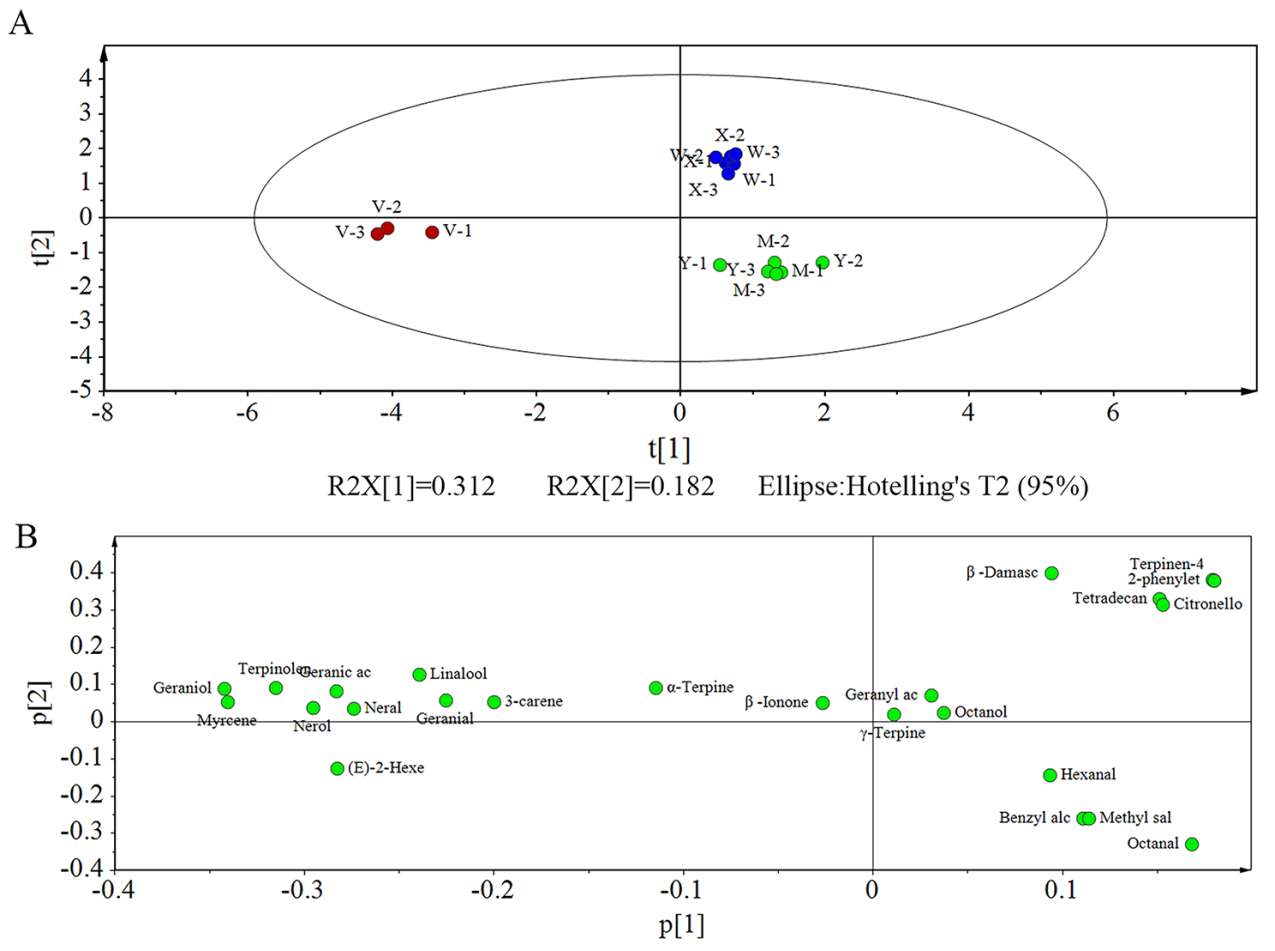
Principal component analysis of volatiles of sample map from skin **(A)** and variable correlation map from skin **(B)**. X stands for ‘Summer Black’, Y stands for ‘Shine Muscat’, M stands for ‘Midnight Beauty, W stands for ‘Centennial Seedless’ and V stands for ‘Victoria’. 1,2 and 3 indicate − 2wrs, 0wrs and 2wrs respectively

The results of the multivariate analysis of the five grape cultivars with different aromas are shown in Fig. 5. Hierarchical cluster analysis of the OVA values of the aroma compounds in the skins revealed that those in the strawberry and neutral types clustered separately. The Shine Muscat and Midnight Beauty muscat types clustered into one group, whereas the Centennial Seedless (muscat aroma) clustered with Summer Black (strawberry aroma). This suggests that the specific aromatic profile of Centennial Seedless remains to be confirmed, possibly because it is a hybrid variety, i.e., the aromatic compounds may have changed somewhat during the hybridization process.

**Fig. 6:**
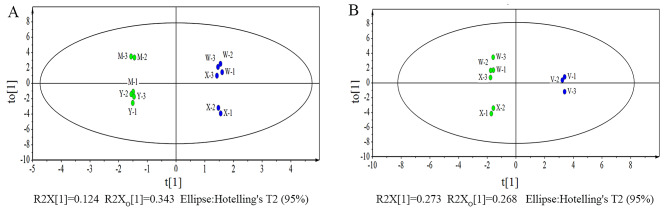
Orthogonal partial least squares-discriminant analysis (OPLS-DA) score plot obtained from the skins of the pairs of the three cultivar aroma types. **(A)** OPLS-DA score plot obtained from the skins of the pairs of the muscat and strawberry aroma types. **(B)** OPLS-DA score plot obtained from the skins of the summer black, centennial seedless and victoria. X stands for ‘Summer Black’, Y stands for ‘Shine Muscat’, M stands for ‘Midnight Beauty, W stands for ‘Centennial Seedless’ and V stands for ‘Victoria’. 1,2 and 3 indicate − 2wrs, 0wrs and 2wrs respectively

To identify the key compounds responsible for differences in aroma, OPLS-DA was conducted between pairs of the three cultivar aroma types, and showed that the samples could be classified effectively by the models (Fig. 6). Significantly different volatiles were selected based on the OVA values of the OPLS-DA model. Table 1 shows that for the various pairs of the three cultivar aroma types, i.e., muscat versus strawberry, muscat versus neutral, and strawberry versus neutral, there were 22, 29, and 22 significantly different volatiles in the skin, respectively. Thus, the volatile profiles of the aroma cultivars agreed with the results obtained from PCA (Fig. 5).

**Table 1:** The significantly different volatiles for the skin of the three aroma types

Muscat VS Strawberry aroma		Muscat VS Neutral		Strawberry VS Neutral	
Trans-2-Hexenal	**+**	Trans-2-Hexenal	+	Trans-2-Hexenal	+
Diisobutyl phthalate	-	Hexanal	-	Hexanal	-
Geraniol	**+**	Octanal	-	Octanal	-
Citronellol	-	1-Nonene	-	1-Nonene	-
Linalool	**+**	Diisobutyl phthalate	-	Diisobutyl phthalate	-
Geranic acid	**+**	Dihydroumbellulone	-	Dihydroumbellulone	-
Phenethyl alcohol	**-**	Benzyl alcohol	-	Benzyl alcohol	-
Beta-Damascenone	**-**	1-Undecanol	-	1-Undecanol	-
4-terpineol	-	2-hydroxy-benzoicacimethylester	-	2-hydroxy-benzoicacimethylester	-
Terpinolene	**+**	Geraniol	+	Geraniol	+
aTerpineol	-	Citronellol	+	Citronellol	+
Terpinen-4-ol	-	Linalool	+	Linalool	+
Myrcene	**+**	Geranic acid	+	Geranic acid	+
Ocimene	**+**	Phenethyl alcohol	+	Phenethyl alcohol	+
(4E,6Z)-2,6-Dimethyl-2,4,6-oct​atriene	**+**	Beta-Damascenone	+	Beta-Damascenone	+
1-Octanol	**+**	4-terpineol	+	4-terpineol	+
Geranyl butyrate	**+**	Terpinolene	+	Terpinolene	+
(E)-3,7-dimethyl-2,6-Octadienal	**+**	aTerpineol	+	aTerpineol	+
3-carene	**+**	Terpinen-4-ol	+	Terpinen-4-ol	+
cis-Geraniol	**+**	Myrcene	+	Myrcene	+
Citral	**+**	Ocimene	+	Ocimene	+
Damascenone	**+**	(4E,6Z)-2,6-Dimethyl-2,4,6-oct​atriene	+	(4E,6Z)-2,6-Dimethyl-2,4,6-oct​atriene	+
		1-Octanol	+		
		Geranyl butyrate	+		
		(E)-3,7-dimethyl-2,6-Octadienal	+		
		3-carene	+		
		cis-Geraniol	+		
		Citral	+		
		Damascenone	+		

The volatiles were selected using OVA values of the OPLS-DA model. + indicated compound contents in the former aroma types was significantly higher than that in the latter aroma types. - indicated compound contents in the former was significantly lower than that in the latter

### Library construction and sequencing

**Fig. 7:**
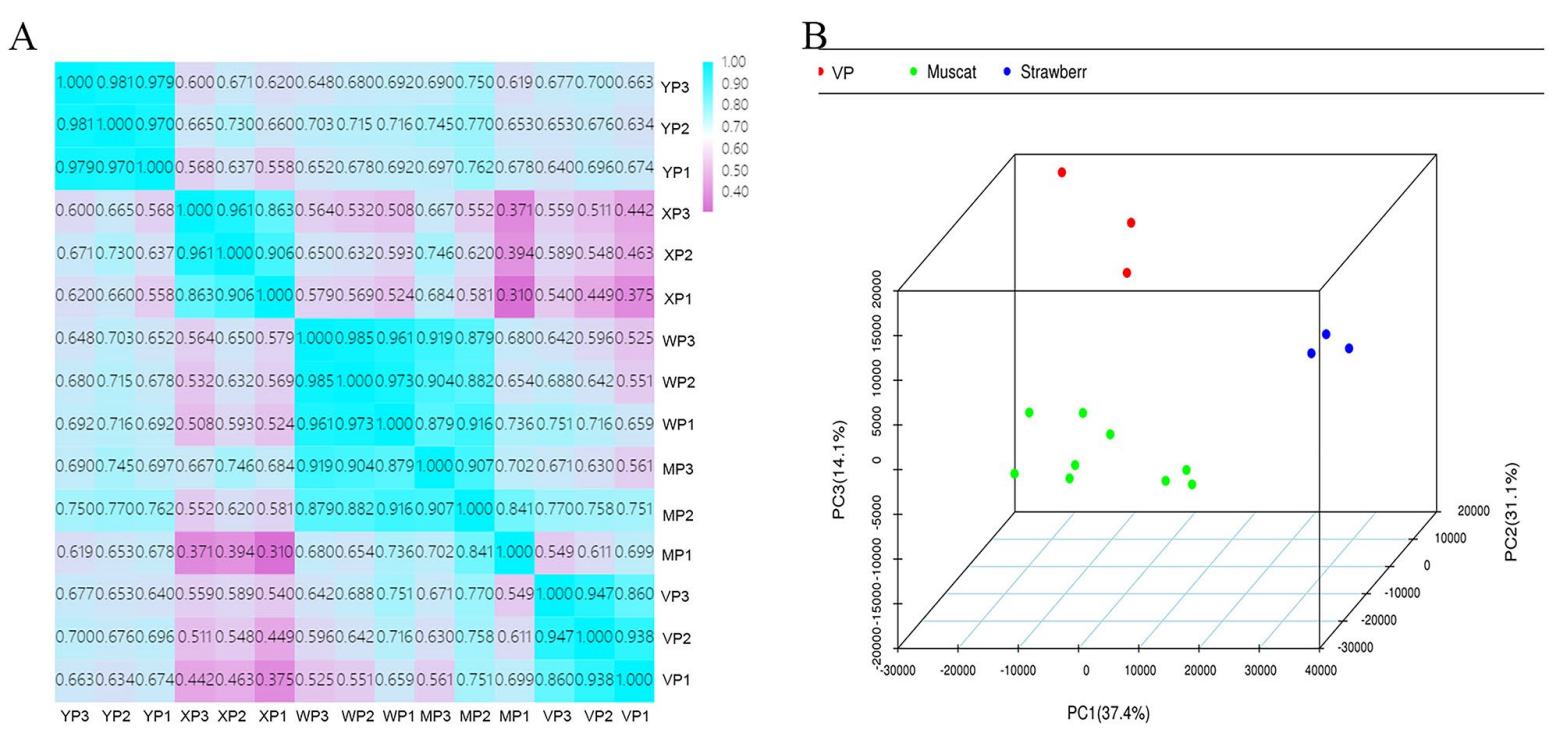
Pearson’s correlation matrix **(A)** and principle component analysis (PCA) **(B)** of genes were performed on three biological replicates of each sample to evaluate correlations and variance between samples

After analysis of the physiological parameters and terpene profiles of the skins and pulps of five cultivars of grapes in the three growth periods, samples in the ripening stage were subjected to RNA-seq gene expression analysis. The HiSeq™ 2500 system (Illumina, San Diego, CA, USA) was used to sequence CK1-3, XP1-3, MP1-3, WP1-3, and YP1-3 cDNA libraries. After rigorous quality checks and data cleaning, a total of 97.67 Gb of clean data was obtained, with each sample having 5.78 Gb of clean data. The percentage of Q30 bases was > 92.69% (Table 2). The expression data of all sequenced samples were then converted into FPKM data and subjected to PCA and Pearson’s correlation analysis (Fig. 7). All samples showed some degree of correlation, with grapes with different aromas in particular showing significant differences (Fig. 7A). However, the correlation was much higher for cultivars with the same aroma. The PCA of the expression profiles of 15 libraries (5 samples with 3 biological replicates each, Fig. 7B) revealed that samples with different aromas could be clearly separated. PC1, PC2 and PC3 explained 37.4%, 31.1%, and 14.1% of the variance, respectively. Thus, the RNA-seq results were confirmed to be highly reliable and suitable for further analyses.

**Table 2:** Summary of sequencing data

Samples	Total Reads	Clean reads	Clean bases	GC Content	%≥Q30	Mapped Reads	Uniq Mapped Reads
VP1	50,568,852	25,284,426	7,530,431,400	47.18%	93.83%	45,633,552 (90.24%)	43,599,912 (86.22%)
VP2	48,708,818	24,354,409	7,254,856,764	46.76%	93.69%	43,844,802 (90.01%)	42,157,420 (86.55%)
VP3	39,402,908	19,701,454	5,864,305,500	46.95%	93.61%	35,551,989 (90.23%)	34,110,102 (86.57%)
XP1	46,092,062	23,046,031	6,897,058,470	47.24%	93.45%	40,457,806 (87.78%)	38,942,133 (84.49%)
XP2	40,978,008	20,489,004	6,107,500,568	47.06%	93.37%	36,322,915 (88.64%)	34,905,010 (85.18%)
XP3	42,872,360	21,436,180	6,410,569,928	47.22%	93.62%	31,089,079 (72.52%)	29,937,835 (69.83%)
MP1	45,320,828	22,660,414	6,779,839,260	46.96%	92.69%	41,062,733 (90.60%)	39,358,371 (86.84%)
MP2	45,780,340	22,890,170	6,829,469,172	46.91%	93.29%	41,866,055 (91.45%)	40,293,175 (88.01%)
MP3	44,622,338	22,311,169	6,673,457,264	47.39%	92.99%	40,476,012 (90.71%)	38,697,323 (86.72%)
WP1	45,275,218	22,637,609	6,761,711,300	47.25%	93.79%	40,463,816 (89.37%)	38,725,485 (85.53%)
WP2	42,709,240	21,354,620	6,384,998,552	46.84%	93.82%	38,579,494 (90.33%)	37,183,611 (87.06%)
WP3	42,514,334	21,257,167	6,340,642,382	46.96%	93.74%	38,224,390 (89.91%)	36,762,952 (86.47%)
YP1	43,896,982	21,948,491	6,267,696,875	46.59%	93.58%	19,599,264 (44.65%)	19,001,467 (43.29%)
YP2	38,649,854	19,324,927	5,781,083,450	46.55%	93.47%	33,208,081 (85.92%)	32,129,115 (83.13%)
YP3	38,848,292	19,424,146	5,790,205,340	46.72%	93.89%	34,066,174 (87.69%)	32,903,856 (84.70%)

1–3: Three biological replicates of skins at ripen-stage; Total reads: original number of reads obtained by sequencing; Clean reads: number of reads after removing low-quality reads and trimming adapter sequences; Clean bases: number of clean reads multiplied by length of clean reads. Q30: Phred score, indicates 99% and 99.9% accuracy of sequenced bases; GC content: percentage of G and C in total bases. Mapped Reads: the number of Reads compared to the reference genome and the percentage of Clean Reads. Uniq Mapped Reads: the number of Reads compared to the unique position of the reference genome and the percentage of Clean Reads

### Identification of DEGs

**Fig. 8:**
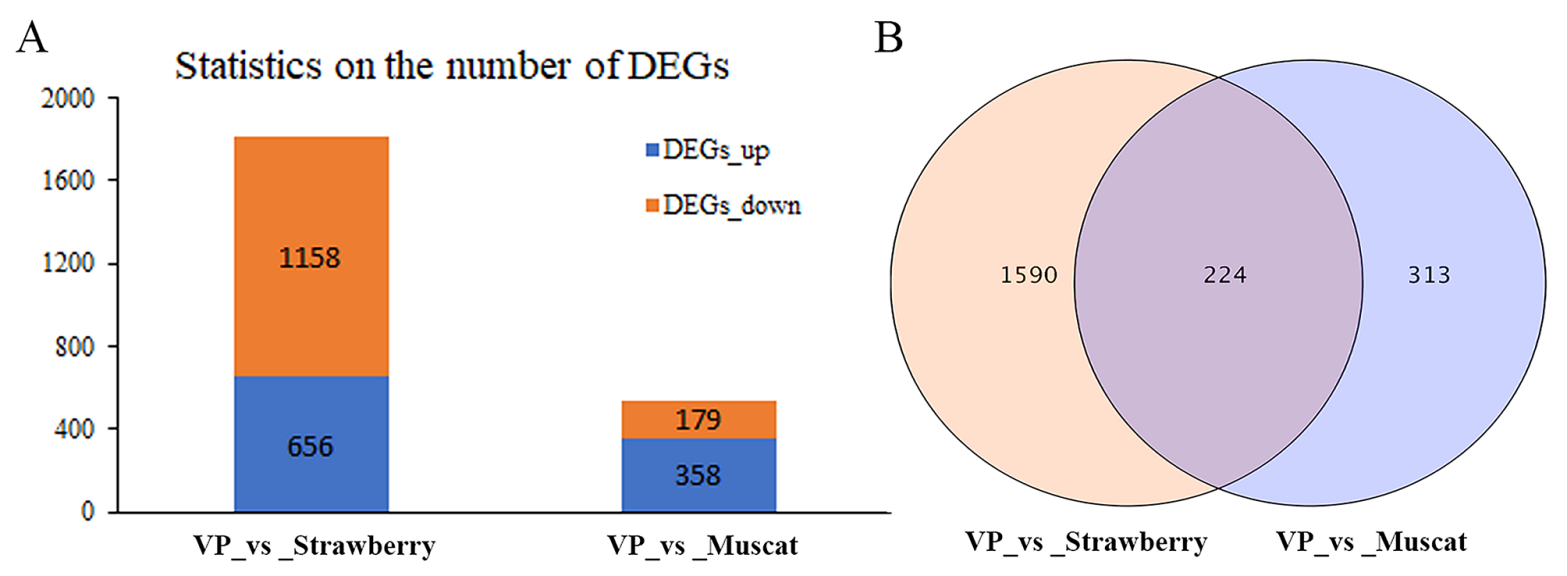
Statistics on the number of DEGs in the skins of grapes with different aromas. **(A)** Statistics on the number of DEGs **(B)** Quantity statistics Venn diagrams of DEGs among the two sampling groups

In this study, gene expression levels were calculated by the FPKM method, and DEGs with log2 (fold change) ≥ 2 and FDR ≤ 0.01 were considered significantly different. There were 1,814 DEGs between neutral and strawberry grapes in the ripening stage, of which 656 DEGs were up-regulated and 1,158 were down-regulated. Of the 537 DEGs between neutral and muscat aroma grapes, 358 were up-regulated and 179 were down-regulated (Fig. 8A). In the figure, the number of DEGs differed considerably between the CK versus strawberry and CK versus muscat comparisons, indicating that the transcript levels of the different aromatic grapes differed considerably at the ripening stage. In addition, we identified 224 common DEGs in the CK versus strawberry and CK versus muscat comparisons (Fig. 8B). This suggests that these DEGs play important regulatory roles in neutral grapes; this finding provides a basis for resolving aroma differences among cultivars.

### Gene annotation and functional classification

An analysis of DEGs between RNA-seq libraries was conducted to identify candidate genes that may be involved in aroma synthesis or metabolism. To functionally annotate the transcriptomes of aroma synthesis or metabolism, 531 unigenes identified in grape skins in the VP versus muscat analysis were compared with six public databases (Supplementary Table S4); in total, 224, 459, 159, 531, 388, and 479 unigenes were annotated in the COG, GO, KEGG, NR, Swiss-Prot, and eggNOG databases, respectively. In total, 1,795 unigenes identified in grape skins in the VP versus strawberry analysis were compared with six public databases, and 854, 1,602, 596, 1,794, 1,383, and 1,685 unigenes were annotated in the COG, GO, KEGG, NR, Swiss-Prot, and eggNOG databases, respectively.

GO enrichment analysis was performed to classify genes into the molecular function, biological process, and cellular categories (Figure S4). A total of 53 functional subcategories were identified for 25,649 genes mapped to different GO function nodes in grape skins with different aromas. The main terms (with the number of associated of genes in parentheses) included cell (21,400), cell part (21,398), cellular process (20,385), metabolic process (19,583), single organization process (19,082), binding (15,658), and catalytic activity (14,225). GO annotations of these genes may be helpful for determining the molecular mechanisms underlying grape skin aroma production and release.

KEGG analysis can reveal DEGs in specific metabolic pathways. To identify relevant genes enriched in metabolic or signalling pathways, DEGs in the ripening stage were mapped to the KEGG database. A total of 5,587 genes were enriched in the KEGG pathway. Among them, 330 genes were annotated to ‘Ribosome’, 311 to ‘Carbon metabolism’, 280 to ‘Starch and sucrose metabolism’, 273 to ‘Plant-pathogen interaction’, 264 to ‘Biosynthesis of amino acids’, 206 to ‘Phenylpropanoid biosynthesis’, and 182 to ‘Amino sugar and nucleotide sugar metabolism’. KEGG enrichment indicated that these pathways play important roles in the aromas of grape skins (Figure S5).

As discussed above, the main plant aroma constituents are terpenoids, aromatic species, and aliphatic compounds. In total, 39 DEGs related to 20 metabolic pathways were identified at the ripening stage (Figure S5), in which the metabolic pathways associated with the biosynthesis of aroma compounds that were more extensively annotated were as follows: ‘Starch and sucrose metabolism’, ‘Biosynthesis of amino acids’, “Phenylpropanoid biosynthesis and metabolism’, ‘Fatty acid metabolism’, and ‘Phenylalanine, tyrosine and tryptophan biosynthesis’.

### Identification of DEGs related to the regulation of aroma metabolism in the ripening stage

**Fig. 9:**
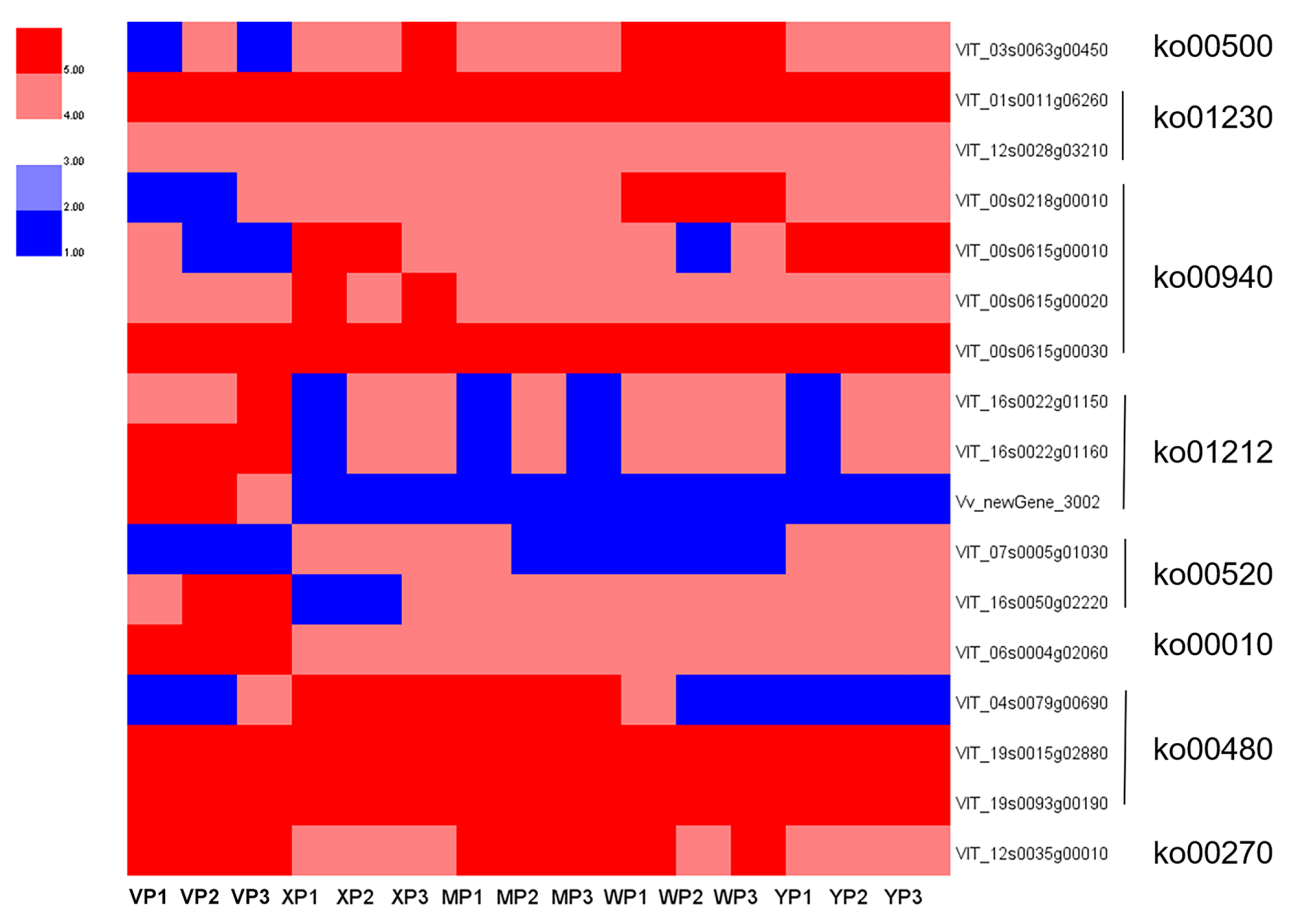
Heatmap plot of DEGs identified by KEGG enrichment analysis at ripen-stages, showing functional categories of significantly over-represented DEGs at ripen-stage. YP stands for ‘Shine Muscat’, MP stands for ‘Midnight Beauty, XP stands for ‘Summer Black’, WP stands for ‘Centennial Seedless’ and VP stands for ‘Victoria’

More detailed comparisons were then made between the neutral and strawberry aroma grape skins, and between the neutral and muscat aroma grape skins. Genes that were significantly differentially expressed between cultivars with different aromas in the ripening stage (Table S3), and the pathway classifications of the enriched genes, are shown in Fig. 9. At this stage, a large number of genes associated with the metabolism of floral volatiles were highly enriched in the ‘Starch and sucrose metabolism’, ‘Biosynthesis of amino acids’, ‘Phenylpropanoid biosynthesis’, ‘Fatty acid metabolism’, ‘Amino sugar and nucleotide sugar metabolism’, ‘Glycolysis/Gluconeogenesis’, ‘Glutathione metabolism’, and ‘Cysteine and methionine metabolism’ pathways, suggesting that regulatory networks control the synthesis of aroma compounds in different aroma grape cultivars.

Combining the GO and KEGG enrichment results revealed128 genes that may be directly involved in the biosynthesis of grape aroma compounds. The biosynthesis of benzene-containing aroma compounds in plants involves the conversion of shikimic acid to phenylalanine, which enables primary and secondary metabolism. Analysis of aroma synthesis and metabolic processes revealed that 4-coumarate: coenzyme A ligase (4CL) and cinnamoyl-CoA reductase (CCR) were mainly enriched in upstream floral aroma synthesis processes. The DEGs included one *VvCYP* (VIT_08s0007g07730), one *VvSDR* (VIT_08s0040g01200), two *VvCCR* (VIT_13s0067g00620, VIT_13s0047g00940), and three *VvADH* (VIT_00s0615g00010, VIT_00s0615g00030, VIT_ 00s0615g00020) genes. Of these genes, only VIT_08s0007g07730 and VIT_13s0047g00940 were highly expressed in neutral grapes; the rest were highly expressed in aromatic grapes.

Furthermore, combining our previous aroma studies with the transcriptome sequencing results of this experiment indicated that one 1-deoxy-d-xylulose-5-phosphate synthase (DXS, VIT_05s0020g02130) gene was differentially expressed among cultivars with different aromas, and high expression was seen in grapes with muscat aroma. Among the other five MEP pathway genes, DXR (1-deoxy-d-xylulose-5-phosphate reductoisomerase, LOC100248516), MCT (2-C-methyl-d-erythritol-4-phosphate cytidyltransferase, LOC100247263), CMK (4-diphosphate cytidyl-2-C-methyl-d-erythritol kinase, LOC100261596), CMS (2-C-methyl-d-erythritol 2,4-cyclodiphosphate synthase, LOC100250076), HDR (4-hydroxy-3-methylbut-2-enyl diphosphate), UDP-glycosyltransferase (UGT, VIT_17s0000g07070, VIT_17s0000g07060), and β-glucosidase (BGLU, VIT_03s0063g02490) were mainly enriched in the downstream stages of floral aroma compound synthesis. However, the gene VIT_17s0000g07060 was highly expressed in neutral grapes.

The final step of terpenoid biosynthesis is catalysis by terpene synthase (TPS) enzymes. TPS expression is usually associated with terpene-rich grapes. However, in this experiment, six *VvTPS* genes were found (VIT_13s0067g00370, Vitis_vinifera_newGene_3216, VIT_13s0067g00380, VIT_13s0084g00010, VIT_00s0271g00010, and VIT_13s0067g00050), the first five of which were highly expressed in grapes with strawberry aroma. VIT_00s0271g00010 was present in grapes with muscat aroma and VIT_13s0067g00050 was expressed in unscented grapes. These results suggest that different genes in the same family have different functions.

Transcription factors (TFs) can control and regulate the expression levels of target genes by binding to promoter regulatory elements. The differential TFs in this experiment and previous studies on grape aroma identified one GATA (VIT_15s0024g00980) and two MYB (VIT_17s0000g07950, VIT_03s0063g02620) TFs, suggesting that these TFs may be important for regulating the biosynthesis of volatile compounds. Furthermore, of the three TFs, only VIT_03s0063g02620 was highly expressed in aromatics, suggesting a negative regulatory role.

### Validation of DEGs by qRT-PCR

**Fig. 10:**
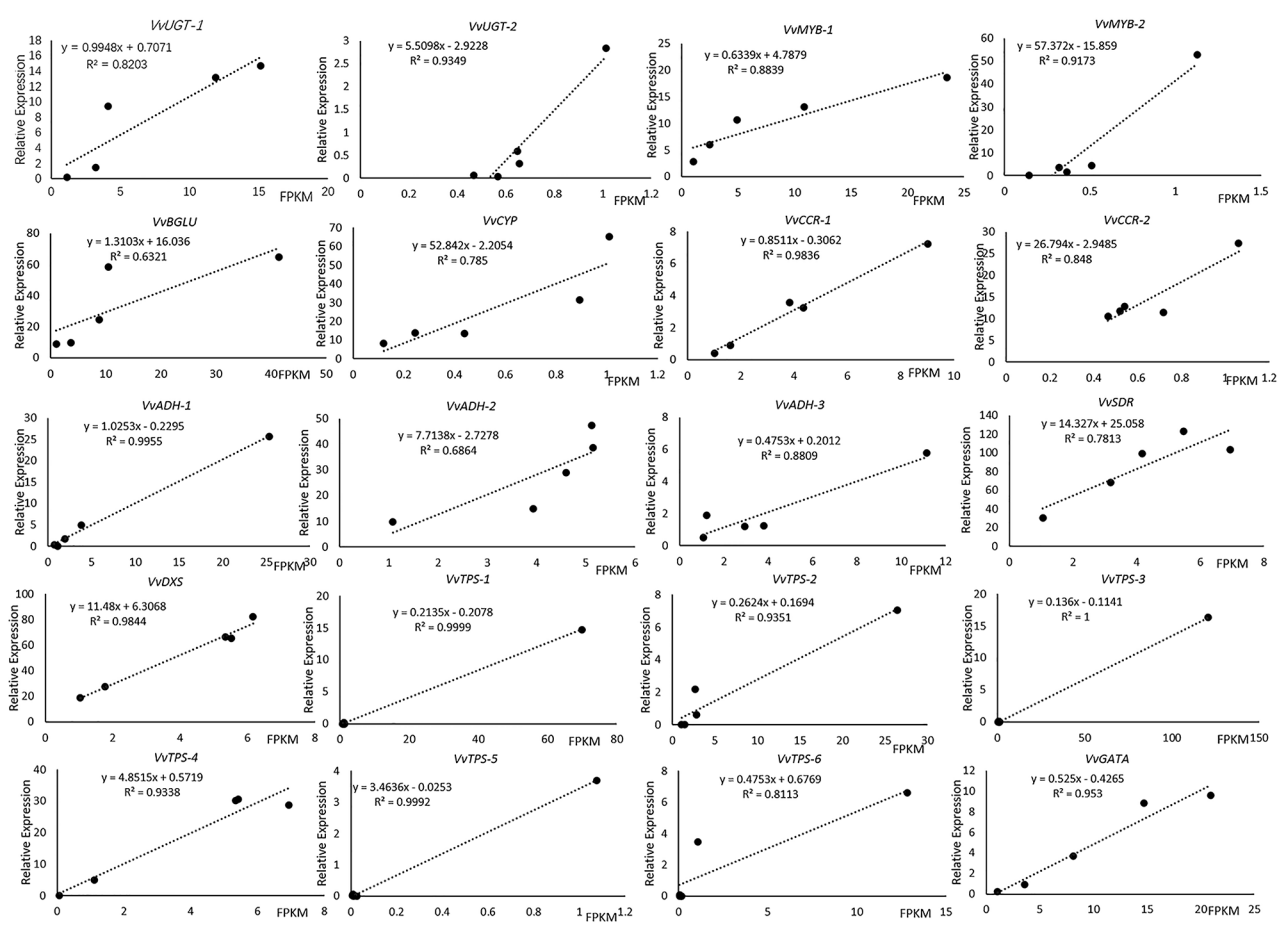
Correlation analysis between qRT-PCR and FPKM at grape ripening stage

To validate the reliability of the RNA-seq data, gene expression analyses of 20 putative terpene biosynthesis genes were performed by qRT-PCR across different cultivars (Fig. 10 and S6). These 20 genes include monoterpene biosynthetic pathway genes (e.g., UGT, BGLU, CYP, CCR, ADH, 17, DXS, and TPS) and TFs (e.g., MYB and GATA). The relative expression levels were generally consistent with the results of deep sequencing. Correlation analysis was used to evaluate the relationship between the RNA-Seq and qRT-PCR. FPKM from RNA-Seq and 2^−ΔΔct^ from qRT-PCR, correlation coefficients near 0.8 were obtained for these gene, respectively, indicating that the RNA-Seq data were reliable.

## Discussion

### The main aroma compounds of different grapes

Grapes, as the second most produced fruit in the world, are widely grown and have an important role in winemaking, due to the differences in flavour among the many cultivars. The distinctive flavour of fresh grapes arises from the balance between sweet and sour tastes in the fruit, and the content of volatile substances. In recent years, identifying the aromatic compounds in grapes has been a key target for studies of aroma [24]. Grape berries contain a large number of volatile components, mostly at low concentrations, and samples are usually pre-treated with suitable concentrations and enrichment methods prior to GC-MS analysis. The SPME technique used in this study combines sampling, extraction, concentration, and injection in a convenient, rapid, solvent-free, selective and sensitive manner [25]. A total of 215 aroma components were quantified in the fruit of five grape cultivars, including the main volatile components such as aldehydes, alcohols, esters, and terpenes. A comparative aroma study revealed that each aroma type has a corresponding characteristic aroma component.

Muscat aroma is closely related to the content of terpenes, the most abundant of which are monoterpenes including linalool, geraniol, nerolidol, citronellol, geranic acid, and α-terpinol [26]. Fenoll [17] et al. studied the type, content, and aroma contribution of monoterpenes during the development of muscat grapes and concluded that linalool was the most important contributor to the aroma. Another study concluded that linalool and geraniol were the main contributors to muscat aroma [27]. The three muscat cultivars in the current study contained significantly higher levels of linalool, geraniol, geranic acid, and terpenes than the other cultivars, with geraniol having the highest average content, followed by geranic acid and linalool. Geraniol was also detected in the skins of ‘Summer Black’; however, its OVA were lower than those of muscat grapes. Thus, geraniol is the most important terpene contributing to the odour of muscat cultivars, but is not a specific component of such cultivars. Linalool and geranic acid are also important active aromatic components in muscat cultivars but, similar to geraniol, they are not unique to such cultivars; they are also detected in strawberry cultivars at concentrations above the threshold.

Strawberry aromas are mainly found in American grape cultivars and hybrids with other species, and the characteristic aroma substances are mainly esters [15, 28]. Linalool and C13-norisoprenoids, ethyl 2-methylbutyrate, and ethyl butyrate are the main aroma-contributing compounds of Summer Black, Vine Ripening, and Giant Peak, respectively [29]. In this study, the odour composition of the strawberry cultivar was complex, with contributions from terpenes, esters, aldehydes, alcohols, and C13-norisoprenoids (mainly trans-2-hexenal in aldehydes, diisobutyl phthalate in esters, alcohols in phenethyl alcohol, β-damascenone in C13-norisoprenoids, and geraniol, citronellol, linalool and geranic acid in terpenoids). Trans-2-hexenal was present in the highest concentration, but also had a high threshold such that the aroma contribution was low. Diisobutyl phthalate, citronellol, and phenethyl alcohol were specific to the strawberry-scented cultivars, and may be responsible for the aroma of ‘Summer Black’ grape. Damascenone had the highest OVA value in the strawberry grape.

Terpenoids and esters are rare in neutral cultivars of grapes; their concentrations tend to be low or almost non-existent, mainly because C_6_ compounds account for the majority of aromatic substances [30]. Oliveira considers hexanal, 2-hexenal, and other C_6_ compounds to be important flavour compounds in grapes. Moreover, they are of great value for evaluating the quality of grapes and determining their origin. In the current study, two alcohols and three aldehydes, along with one ketone and one ester, were the main aroma components of the neutral cultivar, with trans-2-hexenal being the most abundant compound, followed by octanal and hexanal. These substances contributed less to the aroma due to their high threshold values, in agreement with previous studies [29]. Dihydroumbellulone, benzyl alcohol, 1-undecanol, and 2-hydroxy-benzoicacimethylester were also active aroma-presenting components; however, the concentrations were low such that they contributed mainly to the ‘background aroma’.

### RNA-seq analysis of DEGs associated with aroma

Phenylpropanoids volatiles were mainly synthesized from phenylalanine and produced via the shikimate, prephenate, and arogenate synthesis pathways. A number of enzymes were involved in the synthesis of phenylalanine, including CCR, ADH, SDR, and CYP. Notably, SDR genes are involved in many aspects of primary and secondary metabolism, especially in carbonyl-alcohol redox [31]. The SDR family is important for the biosynthesis of a number of compounds derived from phenylalanine, such as 2-phenylethanol (2-PE) in Rosa damascena, which is synthesized from phenylalanine via phenylethylaldehyde as an intermediate [32]. The CYP gene family catalyses a large number of mono-oxidation/hydroxylation reactions in plant primary and secondary metabolism [33]. Therefore, as a component of the benzene pathway, P450 may also have an important influence on the benzaldehyde synthesis pathway [34]. In this study, one *VvCYP* DEG, two *VvCCR* DEGs, three *VvADH* DEGs, and one *VvSDR* DEG were detected in the transcriptome of grapes. *VvCCR* (VIT_13s0047g00940) and *VvCYP* were highly expressed in grapes without an aroma; *Vv*CCR (VIT_13s0067g00620), *VvADH*, and *VvSDR* genes were highly expressed in grapes with an aroma, but their specific regulatory roles are still unknown.

The metabolic pathways of terpenoids are well understood and conserved across species. DXS is the rate-limiting enzyme in the MEP pathway, and phylogenetic analysis of DXSs from other species revealed that this protein is primarily involved in terpenoid synthesis [35]. TPS catalyse the production of monoterpenes using GPP as a substrate, and many monoterpenes undergo hydroxylation, epoxidation, and glycosylation modifications catalysed by reductases, oxidases, or glycosyltransferases (GTs), resulting in a rich variety of monoterpenes [36]. The most important characteristic of TPS genes is their ability to produce multiple terpene compounds of the same type from a single substrate [37]. For example, in Arabidopsis, two TPSs are capable of producing 20 sesquiterpenoids [38]. Phylogenetic studies of TPSs are instructive for identifying unknown functional enzymes. In this study, one *VvDXS* DEG and six *VvTPS* DEGs were identified. When volatile components were analysed in different aromatic grapes, differences in terpenoid composition and content were found between strawberry and muscat aromatic grapes, just as the expression of relevant genes differed among aromatic grapes. *VvDXS* (VIT_05s0020g02130) and *VvTPS* (VIT_00s0271g00010) were highly expressed in muscat grapes. *VvTPS* (VIT_13s0067g00370, Vitis_vinifera_newGene_3216, VIT_13s0067g00380 and VIT_ 13s0084g00010) were highly expressed in the strawberry grape. In contrast, TPS (VIT_13s0067g00050) was highly expressed in the neutral grape. These results suggest that different *VvTPS* genes have different functions, and that these genes (VIT_00s0271g00010, VIT_13s0067g00370, Vitis_vinifera_newGene_3216, VIT_13s0067g00380, and VIT_13s0084g00010) play an important role in terpene synthesis in muscat and strawberry grapes.

### Key genes affecting the volatilization of aroma compounds

GTs are a multi-member gene family, and glycosylation refers to the process of covalent binding of some sugars or non-sugar biomolecules to additional glycosyl groups catalysed by GTs [39]. GTs regulate the biological activity, water solubility, and stability of acceptor molecules and play an important role in the regulation of plant hormone homeostasis, the detoxification of endogenous substances, and modification of defence reactions and secondary metabolites [40]. UGTs are responsible for the conversion of free terpenoids into glycosylated forms [41]. In the current study, we identified two *VvUGT* genes (VIT_17s0000g07070 and VIT_17s0000g07060) potentially involved in the synthesis and volatilization of grape terpenoids by comparative transcriptome analysis.

One DEG (VIT_03s0063g02490) was annotated as BGLU in this study. BGLU is widely expressed in plant tissues and acts mainly in the pre-hydrolysis of aroma compounds, converting aromatic substances in plants from the bound to the free state and releasing aroma volatiles [42, 43]. BGLU significantly increases the aroma concentration [34]. In plum blossoms, *PmBGLU* produces more fragrance, which allows the flowers to release more aromatics [44]. In this study, the high expression of this gene in aromatic grapes was consistent with the grapes having an aroma; this may be the one of the main reasons why more aroma compounds are released from grapes with an aroma.

### Key TFs in the biosynthesis of aroma compounds

Previous studies have shown that TFs known as trans-acting molecules can regulate proteins in gene transcription during plant growth and associated with fragrance synthesis. A variety of TFs were found that could play various roles in the regulation of aroma biosynthesis. The expression profiles of some TFs, including MYB, GATA were also found to be positively related to the monoterpenoid content in this study. MYB TF are commonly in plants. The plant MYB TF family consists of four subclasses: 1R-MYB, R2R3-MYB, R1-MYB, and 4R-MYB. of which R2R3-MYB is the largest class, not only involved in the physiological processes of plant stress resistance, but also responds to JA signals and is an important transcription factor regulating the synthesis of flavonoids and terpenoids [45, 46]. In Petunia, four MYB members regulate benzene volatiles, and the overexpression of MYB genes controls the metabolism of phenylalanine and affects the floral fragrance of the plant [47, 48]. Moreover, the TF *PhMYB* indirectly inhibits the synthesis of coumaric acid derivatives in Petunia [49]. In plum blossoms, the *PmMYB4* gene was significantly more abundant in ‘Xiangxuegongfen’ than in ‘Xiao lve’, and was enriched in of phenolic-containing compounds and phenylpropanoid biosynthesis, which can affect the aroma of plum blossom [44]. GATA TFs play an important regulatory role in the light response, chlorophyll synthesis, and carbon and nitrogen metabolism [50]. GNC, a member of the Arabidopsis GATA TF family, is involved in the regulation of chlorophyll synthesis and glucose signalling, as well as glucose transport [51], which has not been reported in grapes.

In the current study, analysis of transcriptome data showed that two MYB TFs (VIT_17s0000g07950, VIT_03s0063g02620) and one GATA TF (VIT_15s0024g00980) were differentially expressed in grapes with versus without aroma. However, the expression of the three TFs showed opposite trends: VIT_17s0000g07950 and VIT_15s0024g00980 were highly expressed in grapes with an aroma, whereas gene VIT_03s0063g02620 was highly expressed in neutral grapes; this indicates that these TFs are not involved in aroma metabolism. These results also indicate that VIT_17s0000g07950 and VIT_15s0024g00980 are more important in the aroma synthesis pathway in grape. The synthesis of plant terpenoids is a complex process in which a series of enzymes encoded by structural genes are involved in the synthesis of various compounds related to the terpene pathway. Transcription factors specifically bind to recognizable cis-elements in the promoters of structural genes, and ultimately regulate the synthesis of terpenoids by up- or down-regulating the expression of structural genes. However, the regulatory role of TFs in grape volatile compound synthesis needs to be further explored if the molecular mechanisms involved in the biosynthesis of these aromatic compounds are to be fully elucidated.

## Conclusions

This study revealed some differences in the aroma composition and active aromatic substances among neutral, muscat, and strawberry grapes. The neutral grape had the lowest number of aromatic components. The concentration of terpenes was significantly higher in the muscat grapes than in the other aroma cultivars. Diisobutyl phthalate, citronellol, and phenethyl alcohol were unique to strawberry grapes and contributed strongly to its aroma. *VvMYB* and *VvGATA* TFs may play important regulatory roles in the accumulation of aromatic compound biosynthetic precursors in grapes. In addition, the *VvCYP*, *VvCCR*, *VvADH*, *VvSDR*, *VvDXS* and *VvTPS* genesplay important roles in the production and accumulation of aromatic compounds. *VvBGLU* and *VvUGT* genes may be key for controlling the morphology of bound compounds. These results show that volatiles play a key role in the formation of aroma in grape and transcriptome provides a basis for further exploration of the regulatory mechanisms of grape aroma compounds.

## Materials and methods

### Plant material

Based on the aroma characteristics and taste experience as well as the holding of experimental materials, four table grape cultivars, including ‘Summer Black’ (XP), ‘Shine Muscat’ (YP), ‘Midnight Beauty’ (MP), ‘Centennial Seedless’ (WP), which had one of typical aroma types (Muscat, Strawberry), and neutral grape ‘Victoria’ (VP), were collected from a single vineyard (35°18′13.71″ N, 113°55′15.05″ E, Xinxiang, Henan, China). The 5-year-old plants were planted with a spacing of 3 m × 2 m. In the 2019 growing season, samples were collected between 2 weeks before the ripening stage (− 2 wrs) and 2 weeks thereafter (2 wrs). The pulp and skin samples at the three maturity stages (− 2wrs, 0 wrs, and 2 wrs) were used to determine aroma composition and content, and skin samples corresponding to the three maturity stages were used for RNA-sequencing (RNA-seq). For each sample, three bunches of grapes were randomly picked from three vines as one replicate and then transported to the laboratory at 4℃. After standard physicochemical analyses, including of total soluble solids (TSSs), titratable acidity (TA), berry weight, and fruit skin colour, the remaining samples were frozen at − 80°C until the aroma analysis was performed. TSSs were determined using an Abbé refractometer (Rx-5000; Atago, Tokyo, Japan). TA is expressed as grams of tartaric acid per 10 g of fresh weight, as determined by titration with NaOH to a final pH of 8.1 using an automatic titration system [52].

The *L*, *a*, and *b* values of skin colour differences were determined using a CR-400 portable colorimeter (Konica Minolta, Tokyo, Japan), and the colour saturation chroma (C), hue (h), and colour index of red grape (CIRG) values were calculated from these values, as follows:

$$C = {\left( {{a^2} + {b^2}} \right)^{1/2}},$$

$${\rm{h }}{\mkern 1mu} {\rm{ = }}{\mkern 1mu} \,{\rm{arctangent}}\left( {{\rm{b/a}}} \right),$$

$${\rm{CIRG = }}(180 - {\rm{h}}){\rm{/}}\left( {{\rm{L + C}}} \right),$$

where L is the colour brightness, which ranges from 0 to 100, and a* and b* are chromaticity components with values between − 60 and 60. Positive a* represents red, and negative values of a* indicate green; the higher the absolute value, the greater the degree of red or green. A positive value of b* indicates a yellow sample and a negative value of b* indicates a blue sample; a higher absolute value indicates a deeper yellow or blue colour. Specifically, CIRG < 2 for yellow-green, 2 < CIRG < 4 for pink, 4 < CIRG < 5 for red, 5 < CIRG < 6 for dark red, and CIRG > 6 for blue-black.

### Isolation and extraction of free state aroma compounds and the solid-phase microextraction procedure

The samples were prepared according to the method of [53]. About 50 g of the grape samples at different stages of ripeness, with the skin and flesh separated, were removed from the grapes at − 80 °C and placed in liquid nitrogen. The samples were then transferred to an RZ-708 H dry grinder (Royalstar, Guangdong, China); 1 g of polyvinylpyrrolidone and 0.5 g of D-gluconolactone were added, followed by thorough grinding, and the samples were placed in a 50-mL centrifuge tube and left to stand for 4 h at 4 °C. The tube was then quickly placed in a high-speed freezing centrifuge. Each sample was centrifuged at 4 °C for 15 min at 8,000 rev/min, and the top layer of juice was collected and placed in a new 50-mL centrifuge tube. The samples were then stored in a refrigerator at − 80 °C for a short period of time. Three independent replicates of each sample were performed.

The free state-treated sample, 5 mL of ultrapure water, 1 g of NaCl, and 50 µL of 1-octanol were transferred together into a 22mL glass vial. After the vials were capped, the volatiles were equilibrated for 30 min at 60 °C. An extraction head aged at 300℃ for 1 h was then inserted into the vials, 0.8 cm above the liquid level. After the 60-min extraction, the extraction head was removed and inserted immediately into the GC injection port to undergo desorption after 5 min at 280℃.

### Gas chromatography/mass spectrometry analysis

The desorbed volatiles were separated in an Agilent 7890 gas chromatograph (Agilent, Santa Clara, CA, USA) equipped with a DB-5 mass spectrometer (30 m × 0.25 mm × 0.25 μm) (J & W Scientific, Folsom, CA, USA), and coupled with a 5975MS system. The carrier gas was high-purity helium with a flow rate of 1 mL/min. The injector temperature was set at 250℃ in non-divergent mode. The temperature was set to 60℃ for 2 min and then increased to 180℃ at a rate of 5℃/min. Subsequently, the temperature was ramped up to 240℃ at 5℃/min and held at this temperature for 2 min. The ionization voltage was 70 eV, with the source at 230 °C. The quadrupole was set at 150 °C. The acquisitions were performed in full-scan mode over the scanning range of 35–500 m/z. The detected volatiles were identified by comparing spectra and retention times with commercially available NIST 14 libraries. The peak area of each metabolite was normalized using the percentage of the identified component peak area in the area of the internal standard component multiplied by the inner standard mass.

### RNA-seq and transcriptome data analyses

The skins of five cultivars ‘XP’, ‘YP’, ‘MP’, ‘WP’, and ‘VP’ were collected at − 2, 0, and 2 wrs for RNA sequencing. These 15 tissue types were denoted as XP-1, XP-2, XP-3, YP-1, YP-2, YP-3, MP-1, MP-2, MP-3, WP-1, WP-2, WP-3, VP-1, VP-2 and VP-3. Total RNA was extracted using a previously described method [54]. Quantity and quality were checked using the NanoDrop 2000 system (Implen, Westlake Village, CA, USA). RNA integrity was assessed using the RNA Nano 6000 Assay Kit of the Agilent Bioanalyzer 2100 system. The total RNA amount of 1 µg of each library was used as input material for the RNA sample preparations. The NEBNext® Ultra™ RNA library for Illumina (NEB, Ipswich, MA, USA) was used to prepare the kit to generate the sequencing library, and the mRNA was purified from the total RNA using poly-t oligosaccharide-linked magnetic beads. The first-strand cDNA was synthesized by a random hexamer primer and M-MuLV reverse transcriptase. The second-strand cDNA was then synthesized using DNA polymerase I and RNase H. The library fragments were purified using the AMPure XP system (Beckman Coulter, Beverly, MA, USA), and the PCR product was purified using the AMPure XP system (Beckman Coulter). The library quality was evaluated using the Agilent Bioanalyzer 2100 system.

### Mapping of reads to the reference genome, gene annotation, and gene expression analysis

The grape genome and annotations were downloaded from http://plants.ensembl.org/Vitis_vinifera/Info/Index. The clean reads were mapped to the grape reference genome (PN40024 assembly 12X) sequence using Bowtie2 (v2.2.5), and the gene expression level was calculated using RSEM (V1.2.12). The expression quality of unigenes is expressed in terms of fragments per kilobase of exon per million mapped fragments (FPKM). The DESeq R package (1.10.1; R-Forge, Brussels, Belgium) was used to identify DEGs between groups. The p-value was adjusted based on the q-value [55]. A q-value < 0.005 and |log_2_ (fold change) | ≥ 1 were set as the thresholds for significantly differential expression. Gene Ontology (GO) enrichment analysis of the DEGs was performed using the GOseq R packages based on the Wallenius non-central hyper-geometric distribution, which can adjust for gene length bias in DEGs [56]. The Kyoto Encyclopedia of Genes and Genomes (KEGG) is a database providing resources for understanding the high-level functions and utilities of biological systems, such as cells, organisms, and the ecosystem, based on molecular-level information (especially large-scale molecular datasets generated by genome sequencing and other high-throughput experimental technologies;www.kegg.jp/kegg/kegg1.html) [57]. We used KOBAS software to identify DEGs in KEGG pathways [58].

### Real-time quantitative polymerase chain reaction (qRT-PCR) analysis

Twenty genes related to the aroma metabolism in grape skin were analysed using qRT-PCR to verify the accuracy of transcriptome sequencing. The primers for qRT-PCR were synthesized by Sangon Biotech (Shanghai, China); the details are shown in Supplementary Table S5. The expression levels of selected genes were analysed using the LightCycler 480 system (Roche Diagnostics, Indianapolis, IN, USA). The 2^−ΔΔCt^ method was applied to calculate the relative transcript levels of each gene [59]. Three replicates of samples from different plants were collected under the same conditions for qRT-PCR analyses.

### Statistical analysis

All data were subjected to one-way analysis of variance after testing their normality. Orthogonal partial least squares-discriminant analysis (OPLS-DA) and principal components analysis (PCA) were performed using SIMCA14.1; both analyses were weighted using unit variance scaling. Significant differences between cultivars were assessed by Tukey’s post-hoc test (p < 0.05) using SPSS software (ver. 17.0; SPSS Inc., Chicago, IL, USA).

## Electronic supplementary material

Below is the link to the electronic supplementary material.

Supplementary Material 1

Supplementary Material 2

## Data Availability

The transcriptomic data are available in NCBI Gene Expression Omnibus repository (https://www.ncbi.nlm.nih.gov/geo/query/acc.cgi?acc=GSE223965) under accession number GSE223965. The data sets supporting the results of this article are included within the article and its additional files.

## Abbreviations

VOCs: volatile organic compounds
OAVs: odour activity values
MEP: 2-C-methyl-d-erythritol-4- phosphate
XP: Summer Black
YP: Shine Muscat
MP: Midknight Beauty
WP: Centennial Seedless
VP: Victoria
WRS: weeks ripen-stage
IPP: isopentenyl diphosphate
DMAPP: dimethylallyl diphosphate
DXS: 1-deoxy-d-xylulose-5-phosphate synthase
DXR: 1-deoxy-d-xylulose-5-phosphate reductoisomerase
HDR: hydroxymethylbutenyl diphosphate reductase
HMGS: 3-hydroxy-3-methylglutaryl-CoA synthase
HGMR: HGM-CoA reductase
TPS: terpenoid synthase
SPME: solid-phase micro-extraction
GC–MS: gas chromatography–mass spectrometry
RNA-seq: mRNA sequencing
TSSs: total soluble solids
TA: titratable acidity
TFs: transcription factors
DEGs: differentially expressed genes
GO: gene ontology
KEGG: Kyoto Encyclopedia of Genes and Genomes
ORFs: open reading frames
PCA: principle component analysis
FPKM: fragments per kilobase per million mapped reads
UGT: UDP glycosyltransferase
BGLU: β-Glucosidase
CCR: cinnamoyl-CoA reductase
4CL: 4-coumarate: coenzyme A ligase
CAD: cinnamyl alcohol dehydrogenase
