# Supplementary material for: Comprehensive study of volatile compounds and transcriptome data providing genes for grape aroma
Source: BMC Plant Biol. 2023 Mar 31;23:171. doi: 10.1186/s12870-023-04191-1 (PMC10064686; doi:10.1186/s12870-023-04191-1)
Supplement: Supplementary file 1 — Supplementary Material 1 [file 12870_2023_4191_MOESM1_ESM.docx]

Table S1 Content and number of total volatile compounds in the skin of five grape cultivars at different stages of development (μg^.^kg^-1^)

| Cultivars  Content  (μg^.^kg^-1^) | Shine Muscat | | | Midknight Beauty | | | Summer Black | | | Centennial seedless | | | Victoria | | |
| --- | --- | --- | --- | --- | --- | --- | --- | --- | --- | --- | --- | --- | --- | --- | --- |
| Skin | skin-1 | skin-2 | skin-3 | skin-1 | skin-2 | Skin-3 | skin-1 | skin-2 | skin-3 | skin-1 | skin-2 | Skin | skin-1 | skin-2 | skin-3 |
| Alcohols | 5.11±0.07  （1） | 0  （0） | 7.40±0.13  （1） | 0.61±0.06  （1） | 9.06±0.43  (2) | 5.11±0.23  （1） | 11.86±0.87（1） | 7.20±1.02（3） | 0.30±0.05  (1) | 1.48±0.11  (2） | 6.53±0.18  （6） | 1.03±0.02  (1) | 4.7±0.61  (1) | 5.08±0.13 （4） | 14.00±0.99 (5) |
| Esters | 26.07±1.23（8） | 21.06±2.65（8） | 8.46±0.17  （6） | 6.62±0.19  （7） | 6.54±0.14  （5） | 4.46±0.18  （4） | 18.63±1.02  (4) | 14.94±0.87（7） | 9.02±0.22  (9) | 19.87±1.08（8） | 13.82±0.56（5） | 18.42±1.22  (7) | 16.89±0.77  (7) | 23.40±1.55（13) | 6.77±0.12  (7) |
| Aldehydes | 85.51±3.52（2） | 79.89±2.73（1） | 60.70±2.07（1） | 96.60±2.75  （3） | 122.05±5.31  （3) | 114.49±3.44（4） | 43.01±1.65  (1) | 79.04±2.78（1） | 65.66±2.05（2） | 62.72±2.05（1） | 74.39±3.78（2） | 81.22±3.89  (3) | 67.34±2.63  (4) | 70.05±2.78  (2) | 71.52±2.77 (2) |
| Terpenes | 46.89±1.38（6） | 87.33±4.75（6） | 96.19±5.23（9） | 292.17±5.33（14) | 625.10±10.38  (11) | 584.20±9.78 (13) | 100.65±2.64  (9) | 92.77±2.78（11) | 267.13±3.85（14） | 353.03±5.43（13） | 334.27±4.89（11) | 361.60±3.43 (12) | 4.73±0.23  (1) | 5.16±0.55  (4) | 4.62±0.38  (9) |
| C13-Norisoprenoids | 2.53±0.12  （2） | 2.64±0.08  （2） | 1.67±0.05  (1) | 0.89±0.02  (1) | 1.29±0.01  (1) | 2.31±0.12  (2) | 7.03±0.13  (2) | 5.16±0.22  (2) | 2.94±0.13  （2） | 4.01±0.25  （2） | 0.86±0.12  (1) | 5.09±0.17  (2) | 1.47±0.11  (1) | 0.78±0.05  (1) | 0.71±0.03  (1) |
| Ketones | 2.16±0.35  （2） | 1.92±0.16  （3） | 0  (0) | 5.12±0.22  (2) | 0  (0) | 2.15±0.14  (3) | 2.36±0.18  (2) | 9.74±0.72  (5) | 0.26±0.01  （2） | 1.62±0.13  （2） | 3.66±0.04  (2) | 3.43±0.33  (3) | 7.14±0.75  (3) | 0.98±0.18  (2) | 0.57±0.03  (1) |
| total | 168.28±6.87 (21) | 192.84±6.35（20） | 174.43±5.83 (18) | 402.01± 6.86 (28) | 764.04±13.78（22） | 712.72±12.65 (27) | 140.96±4.98 (19) | 208.86± 6.35 (29) | 345.32±5.23（30） | 442.73±8.08（28） | 433.54±7.65  (27) | 470.80±8.02 (28) | 102.27±3.12 (17) | 105.46±3.43 (26) | 98.19± 2.78 (25) |

Note: The numbers in the top row of the table indicate the amount of total volatile compounds in the skin at the time of development and the numbers in the bottom row indicate the number of types of total volatile compounds. Mean ± standard error (n=3). 1,2 and 3 indicate -2wrs, 0wrs and 2wrs respectively.

Table S2 Content and number of total volatile compounds in the pulp of five grape cultivars at different stages of development (μg^.^kg^-1^)

| Cultivars  Content  (μg^.^kg^-1^) | Shine Muscat | | | Midknight Beauty | | | Summer Black | | | Centennial seedless | | | Victoria | | | |
| --- | --- | --- | --- | --- | --- | --- | --- | --- | --- | --- | --- | --- | --- | --- | --- | --- |
| Compounds | pulp-1 | pulp-2 | pulp-3 | pulp-1 | pulp-2 | pulp-3 | pulp-1 | pulp-2 | pulp-3 | pulp-1 | pulp-2 | pulp-3 | pulp-1 | pulp-2 | pulp-3 |  |
| Alcohols | 2.62±0.03  (2) | 3.85±0.35  (1) | 2.75±0.03  (2) | 7.2±0.32  (2) | 1.24±0.05  (1) | 1.75±0.14  (2) | 5.37±0.33（6） | 0  （0） | 2.16±0.12  （2） | 10.33±0.79  (4) | 6.31±0.25  (2) | 5.86±0.27  (6) | 6.63±0.33  (5) | 3.92±0.18  (4) | 2.5±0.11  (3) |  |
| Esters | 7.12±0.55  (9) | 12.54±0.78  (11) | 14.51±0.88  (10) | 11.87±0.67  (9) | 11.17±0.56  (8) | 4.11±0.33  (7) | 7.54±0.67  （7） | 22.06±1.45（13） | 24.01±1.58（12) | 8.33±0.66  (6) | 14.92±0.87  (11) | 13.97±0.84  (11) | 8.31±0.65  (8) | 18.72±1.08  (13) | 13.96±0.84(10) |  |
| Aldehydes | 0.39±0.08  (1) | 0.41±0.07  (1) | 0  (0) | 0  (0) | 13.4±0.65  (1) | 12.92±0.58  (1) | 11.69±0.48（1） | 17.98±0.87（1） | 14.11±0.65  (2) | 0  (0) | 15.73±0.55  (1) | 10.03±0.45  (1) | 15.52±0.56  (1) | 9.21±0.52  (1) | 0  (0) |  |
| Terpenes | 9.67±0.65  (3) | 14.91±0.73  (5) | 13.13±0.52 (3） | 16.58±0.68  (5) | 12.45±0.45  (2) | 40.88±2.57  (4) | 4.56±0.45（4） | 10.77±1.03（3） | 2.21±0.05  (3) | 23.68±2.15  (3) | 6.37±0.55  (4) | 3.39±0.31  (4) | 0  (0) | 6.48±0.62  (3) | 1.31±0.02  (1) |  |
| C13-Norisoprenoids | 0.87±0.01  (1) | 1.27±0.13  (2) | 0.51±0.01  （1） | 4.25±0.25  (2） | 3.57±0.18  (2) | 3.90±0.21  (2) | 3.37±0.23（2） | 2.58±0.25（1） | 3.34±0.14  (2) | 3.09±0.45  (2) | 3.19±0.45  (2) | 0.60±0.02  (1) | 1.66±0.03  (2) | 0.45±0.01  (1) | 1.94±0.08  (2) |  |
| Ketones | 0.98±0.02  (1) | 5.66±1.04  (2) | 0.98±0.21  （3） | 1.28±0.05  （1） | 2.09±0.12  (2) | 3.97±0.23  （3） | 0.90±0.06（2） | 0.91±0.04（2） | 0.82±0.04  (1) | 3.32±.0.78  (2) | 5.71±1.02  (4) | 0.75±0.11  (1) | 1.11±0.04  (3) | 1.05±0.03  (3) | 0.89±0.02  (2) |  |
| total | 21.65±1.05  (17) | 38.63±2.32  (22) | 31.88±1.23（19） | 41.18±1.82  (19) | 43.92±1.25  (16) | 67.54±3.85（19） | 33.43±1.83（22） | 54.3±3.25（20） | 46.61±2.11  (22) | 48.75±3.23  (17) | 52.32±3.28  (24) | 34.60±1.86  (24) | 33.23±1.24  (19) | 39.83±2.12  (25) | 20.61±0.83 (18) |  |

Note: The numbers in the top row of the table indicate the amount of total volatile compounds in the pulp at the time of development and the numbers in the bottom row indicate the number of types of total volatile compounds. Mean ± standard error (n=3). 1,2 and 3 indicate -2wrs, 0wrs and 2wrs respectively.
