# Supplementary material for: Comprehensive study of volatile compounds and transcriptome data providing genes for grape aroma
Source: BMC Plant Biol. 2023 Mar 31;23:171. doi: 10.1186/s12870-023-04191-1 (PMC10064686; doi:10.1186/s12870-023-04191-1)
Supplement: Supplementary file 2 — Supplementary Material 2 [file 12870_2023_4191_MOESM2_ESM.docx]

**Supplementary Materials**


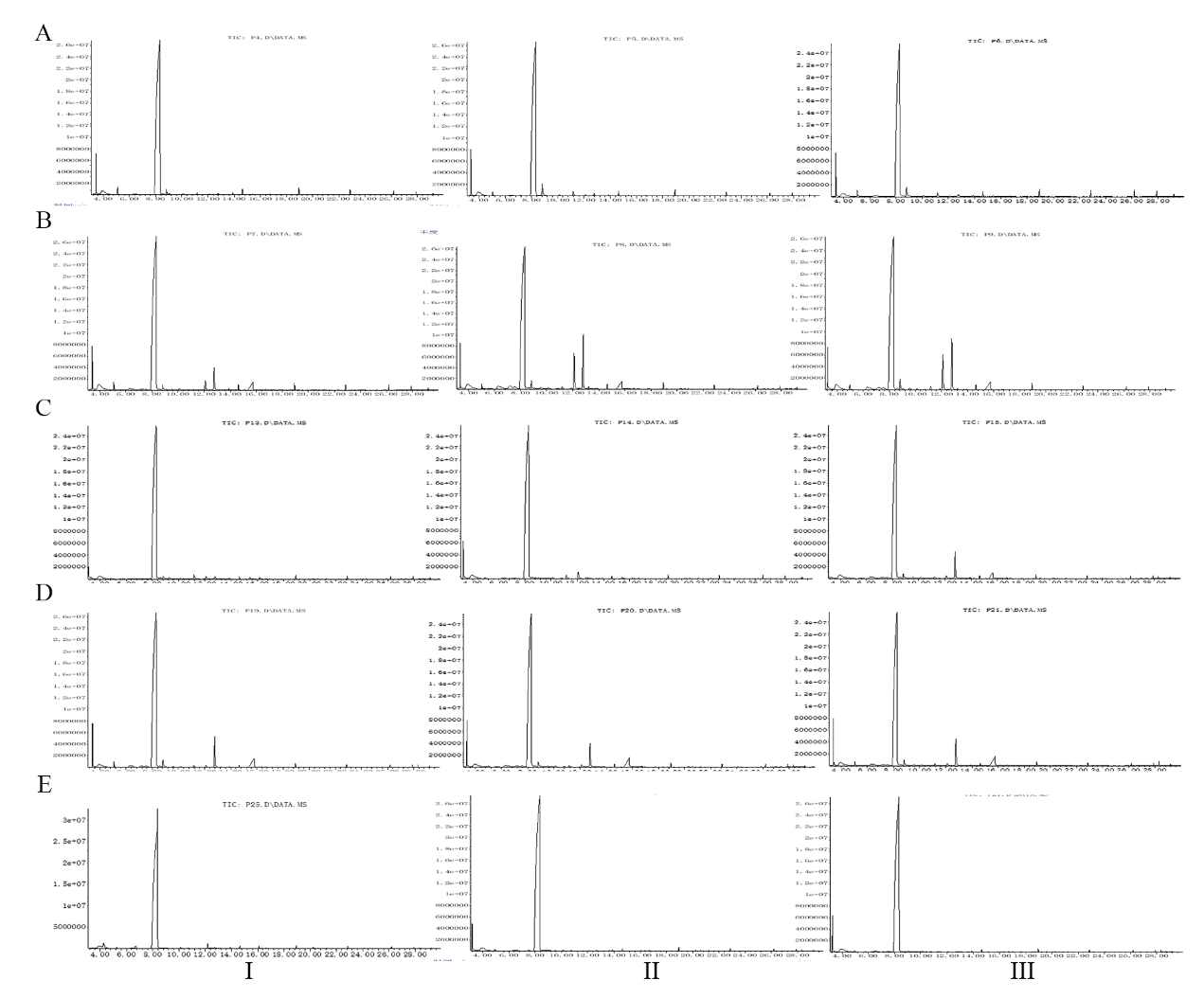


Fig.S1 GC-MS analysis of volatile aroma components in the skins of five cultivars grape with ion flow diagrams

A-E represent Shine Muscat, Midknight Beauty, Summer Black, Centennial seedless and Victoria, respectively. I, II and III indicate -2wrs, 0wrs and 2wrs respectively.


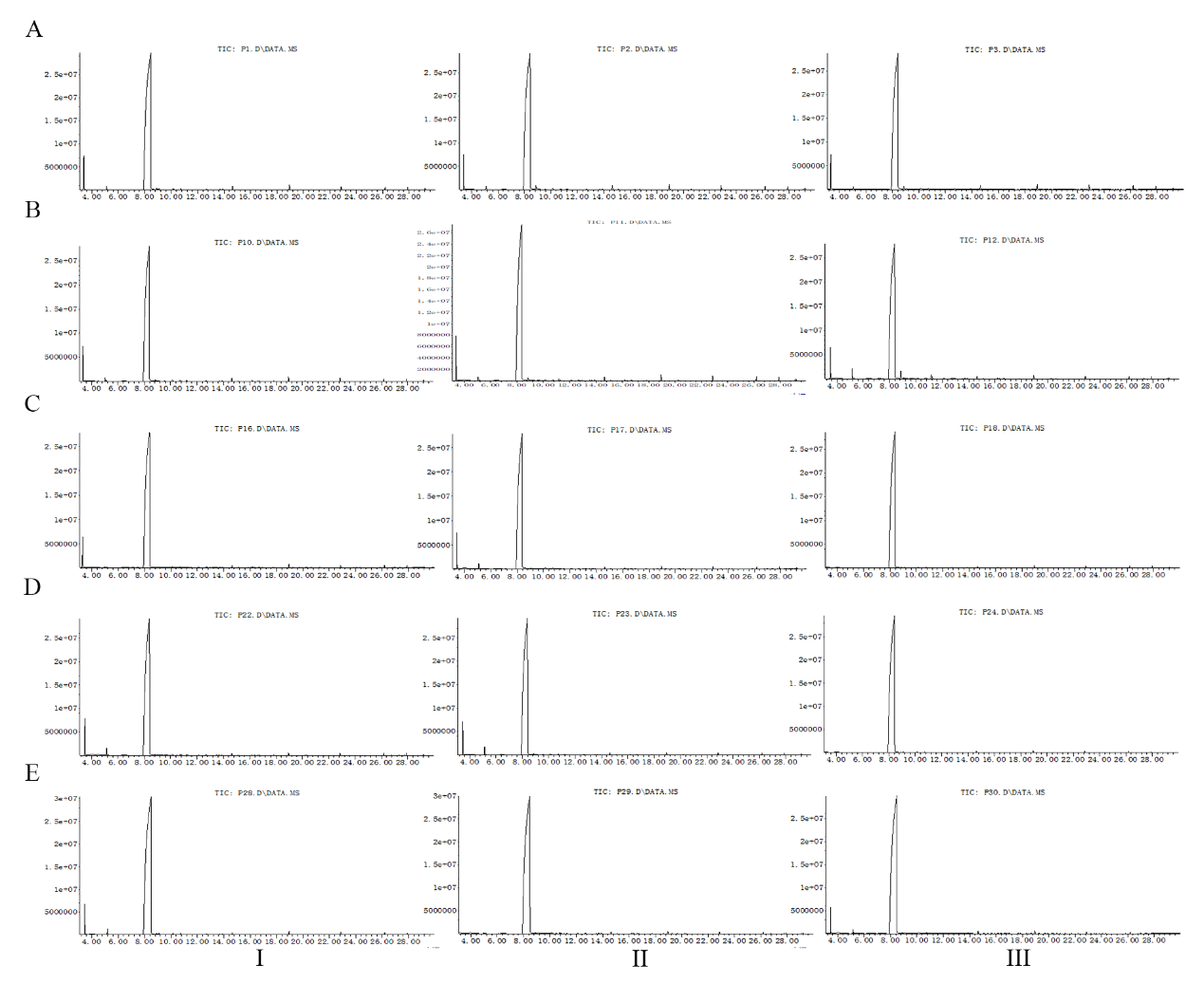


Fig.S2 GC-MS analysis of volatile aroma components in the pulps of five cultivars grapes with ion flow diagrams

A-E represent Shine Muscat, Midknight Beauty, Summer Black, Centennial seedless and Victoria, respectively. I, II and III indicate -2wrs, 0wrs and 2wrs respectively.


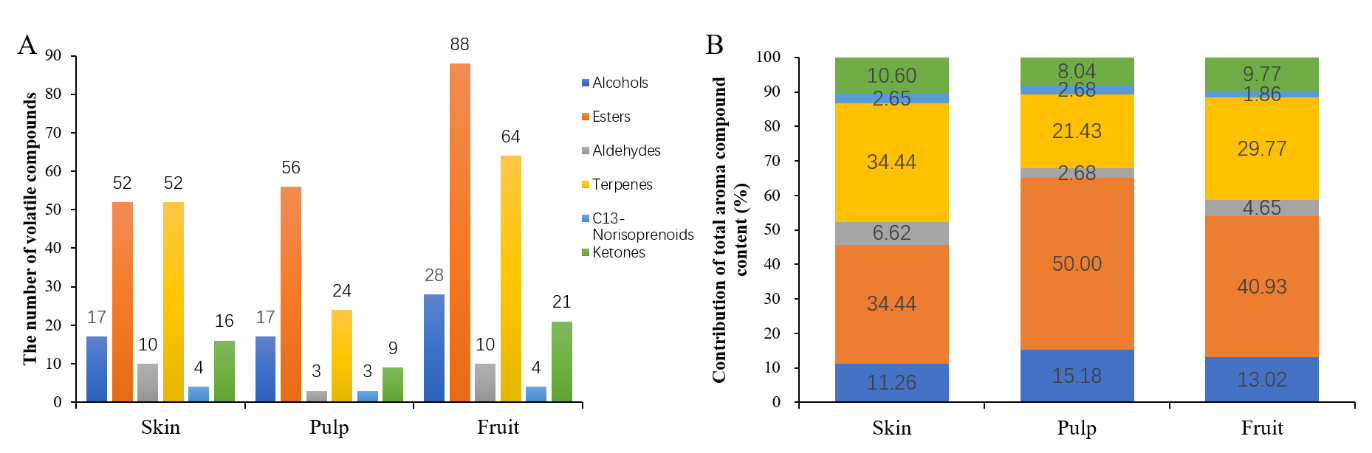


Fig. S3 Composition and quantitative statistics of volatile compounds in skin, pulp and fruit

A: Types and amounts of volatile compounds in skin, pulp and fruit. B: contribution ratio of total aroma substance content in skin, pulp and fruit.


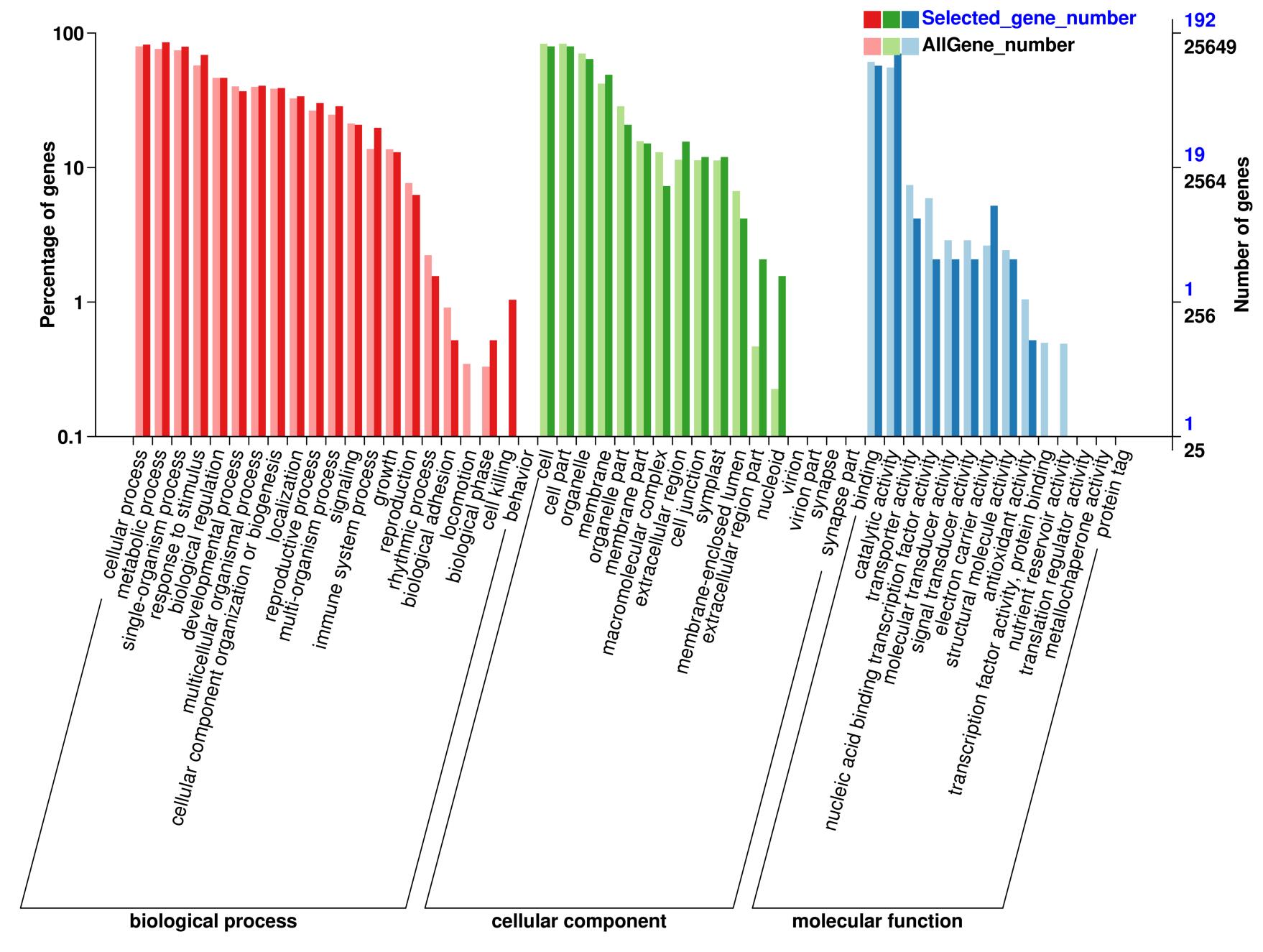


Fig. S4 Gene ontology pathway enrichment of the genes at the ripen-stages of neutral, muscat and strawberry aroma grape skins.


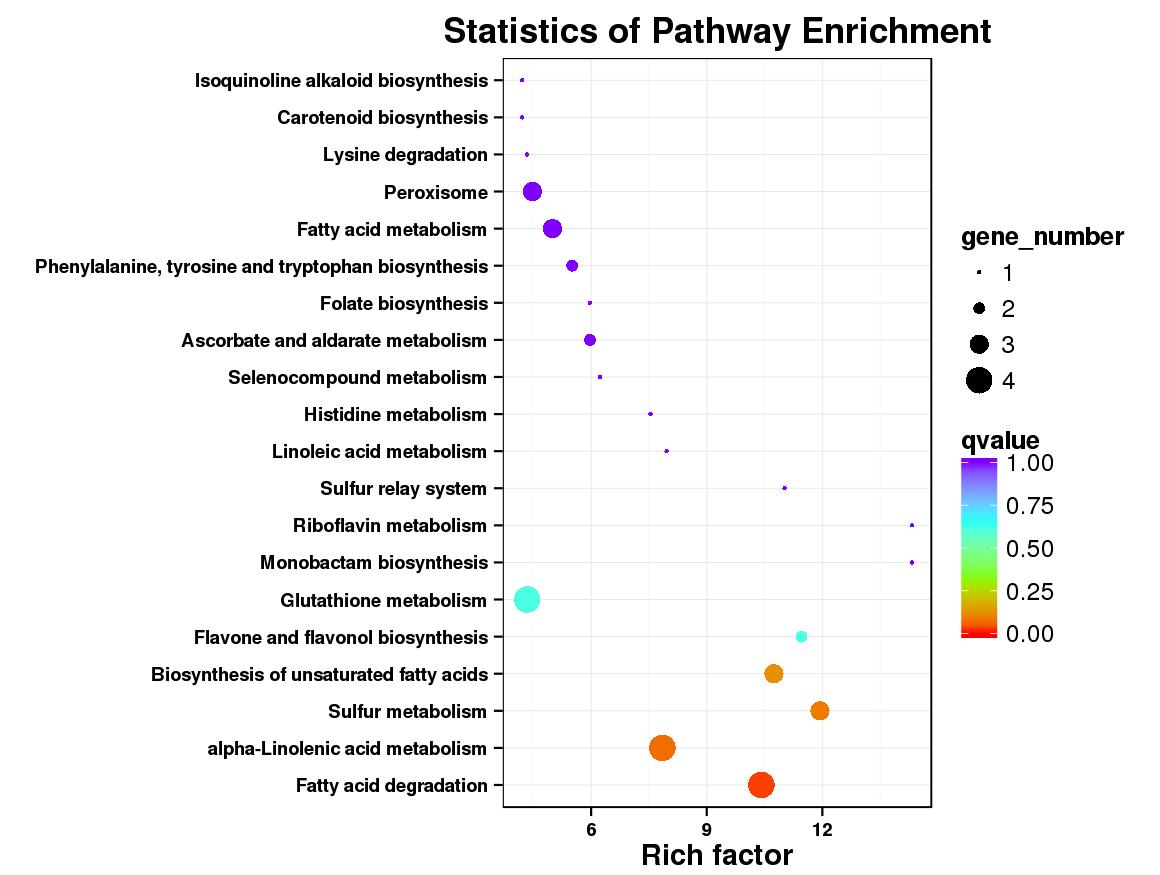


Fig.S5 KEGG pathway enrichment of the genes at the ripen-stages of neutral, muscat and strawberry aroma grape skins (www.kegg.jp/kegg/kegg1.html).


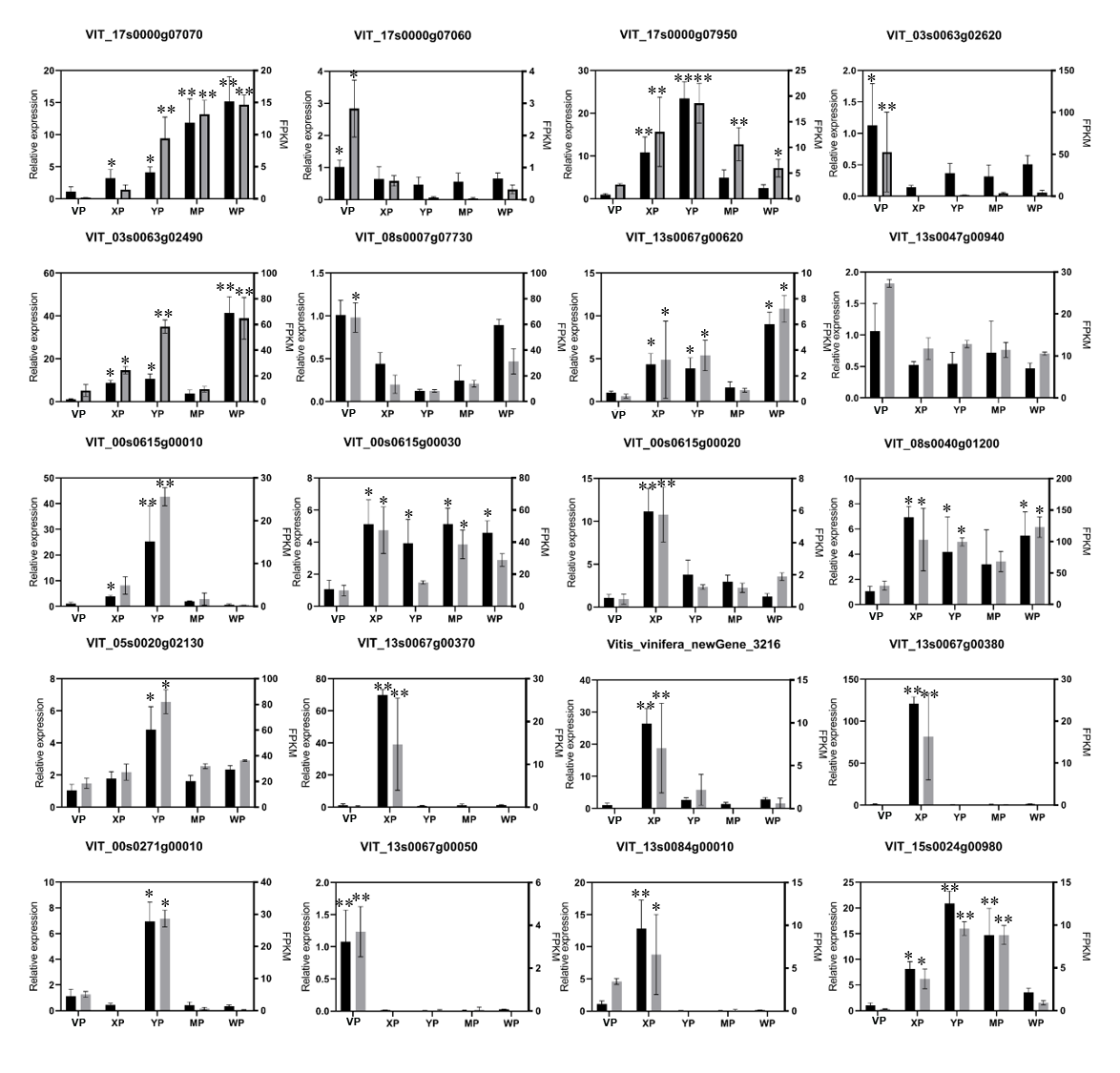


Fig.S6 qRT-PCR validation of genes related to grape aroma compound biosynthesis in different cultivars.

YP stands for ‘Shine Muscat’, MP stands for ‘Midnight Beauty, XP stands for ‘Summer Black’, WP stands for ‘Centennial Seedless’ and CK stands for ‘Victoria’. Black bars represent relative expression. grey bars represent FPKM. Significant differences at P_0.05_ and P_0.01_ levels are marked by* and **, respectively

Table S3 The primers for qRT-PCR

| Gene ID | Forward | Reverse |
| --- | --- | --- |
| VIT_17s0000g07070 | TTTTAGCTCACCCTTCTGTTG | CCACCTTCCATTTGTCGCATA |
| VIT_17s0000g07060 | GCTGATACAACAGTTGGGTG | AAGCCTTGGAATGTGAAATA |
| VIT_17s0000g07950 | CTCCTCGAACTGCAACCAGAG | GAAACTGAGCCCTCCATGTCC |
| VIT_03s0063g02620 | TCTGGCTCATCCTGGACTGC | TTCACTTCCTCCACGGTTTT |
| VIT_03s0063g02490 | CTCGGGCAACAATATCAAGTAC | CACCCTGTCTCAGCAACAACA |
| VIT_08s0007g07730 | CCTCACCAATCCCTTCACAA | AGCCGTAGGTTAGGTAATCAGCAT |
| VIT_13s0067g00620 | GTATGTGCTTTCAAAGACGCTA | AATTGATTGTTGGCTGTAGGG |
| VIT_13s0047g00940 | AGAGGCAGATACTGTTTAGTAGGG | CTGGTTCATAAGGCTTGTCGT |
| VIT_00s0615g00010 | GCAAGAAGAAGGAAGCCATAGAA | GGAAGAAGAGGGTGAACAGCAGA |
| VIT_00s0615g00030 | GATTGTAGGCATAGTGACAGAGG | GTGGTGGTTCCATCATAGTAAGTG |
| VIT_00s0615g00020 | AAAGTTGGTGTTGGGTGTCTG | GTGGTGGTTCCATCATAGTAAGTG |
| VIT_08s0040g01200 | AGAGCGAATGCAATAGCACCT | AATCAAGTATCGCACCAGGGA |
| VIT_05s0020g02130 | CAGAAGCAGAGGTGGACAAGG | ATCCCAACATCAAAGCATCGT |
| VIT_13s0067g00370 | AGATGTTGTTGACAGGTGGGATA | AAGCCATTTCATTAGTGGAGTTGTA |
| Vitis_vinifera_newGene_3216 | TCACAAAGGTCTTGGCACTG | ATCCCATCTGTCAATAGCATCT |
| VIT_13s0067g00380 | GATGTTGTTGACAGGTGGGATA | AAGCCATTTCATTAGTGGAGTT |
| VIT_00s0271g00010 | ATCCCAGATCCAAGGTTGTCAGA | GAGTGAGTTCATCAAGGGTTCCA |
| VIT_13s0067g00050 | AAGAAGACGCCCGTAAACAC | GGAATGGGCTCAATGAGTAG |
| VIT_13s0084g00010 | AGGAGAAAGTACCTTGGAGCAG | GAACCACCTTGCCTCTAACC |
| VIT_15s0024g00980 | AATATCTAAGCCGCCTCCTG | GCTCGGTGACTGACTGGTAA |
| βactin-F | TTCTCGTTGAGGGCTATTCCA | CCACAGACTTCATCGGTGACA |

Table S4 Statistics on the number of DEGs with annotations

| DEG Set | Total | COG | GO | KEGG | NR | Swiss-Prot | eggNOG |
| --- | --- | --- | --- | --- | --- | --- | --- |
| CK_vs_Muscat | 531 | 224 | 459 | 159 | 531 | 388 | 479 |
| CK_vs_strawberr | 1795 | 854 | 1,602 | 596 | 1,794 | 1,383 | 1,685 |

Table S5 FPKM of different genes in different aromatic grapes at ripening stage

| Gene Name | CK_FPKM | XP_FPKM | MP_FPKM | WP_FPKM | YP_FPKM |
| --- | --- | --- | --- | --- | --- |
| VIT_03s0063g00450 | 0.04±0.01 | 3.37±0.44 | 0.23±0.09 | 6.50±1.66 | 2.23±0.37 |
| VIT_01s0011g06260 | 21.10 ±1.15 | 56.56±9.15 | 39.41±9.62 | 37.68±2.78 | 57.38±1.65 |
| VIT_12s0028g03210 | 0.48 ±0.20 | 2.19±0.92 | 2.24±0.77 | 3.48±1.25 | 1.05±0.46 |
| VIT_00s0218g00010 | 0.03 ±0.05 | 3.15±0.63 | 0.58±0.12 | 7.87±3.30 | 0.25±0.07 |
| VIT_00s0615g00010 | 0.02 ±0.04 | 4.89±2.04 | 1.71±0.43 | 0.11±0.18 | 25.60±2.11 |
| VIT_00s0615g00020 | 0.49 ±0.32 | 5.76±1.71 | 1.19±0.29 | 1.89±0.24 | 1.24±0.14 |
| VIT_00s0615g00030 | 9.87 ±3.27 | 47.43±14.51 | 38.56±8.87 | 28.88±3.96 | 14.91±1.03 |
| VIT_16s0022g01150 | 4.02 ±0.86 | 0.21±0.01 | 0.04±0.01 | 0.43±0.15 | 0.45±0.09 |
| VIT_16s0022g01160 | 11.78 ±1.95 | 0.23±0.04 | 0.02±0.01 | 1.13±0.45 | 0.13±0.01 |
| Vv_newGene_3002 | 5.76 ±1.36 | 0 | 0 | 0 | 0 |
| VIT_07s0005g01030 | 0 | 1.65±0.81 | 0 | 0 | 2.16±0.24 |
| VIT_16s0050g02220 | 13.25 ±8.10 | 0.02±0.01 | 1.06±0.19 | 0.91±0.52 | 0.53±0.06 |
| VIT_06s0004g02060 | 11.31 ±2.86 | 0.32±0.16 | 1.69±0.80 | 0.46±0.32 | 1.02±0.13 |
| VIT_04s0079g00690 | 0.02 ±0.01 | 852.20±692.17 | 504.90±196.56 | 0.16±0.28 | 0 |
| VIT_19s0015g02880 | 3126.54 ±434.76 | 733.00±151.19 | 179.06±10.77 | 996.54±161.33 | 167.72±19.36 |
| VIT_19s0093g00190 | 222.95 ±84.07 | 2203.95±528.96 | 454.77±75.96 | 653.01±139.63 | 483.77±37.00 |
| VIT_12s0035g00010 | 26.00 ±11.63 | 1.62±0.78 | 7.96±3.91 | 5.76±1.27 | 2.58±1.23 |
